# Site and Bioenergy Cropping System Similarly Affect Distinct Live and Total Soil Microbial Communities

**DOI:** 10.3389/fmicb.2021.725756

**Published:** 2021-10-14

**Authors:** Sarah I. Leichty, Christopher P. Kasanke, Sheryl L. Bell, Kirsten S. Hofmockel

**Affiliations:** ^1^Earth and Biological Sciences Directorate, Pacific Northwest National Laboratory, Richland, WA, United States; ^2^Department of Agronomy, Iowa State University, Ames, IA, United States

**Keywords:** switchgrass, maize, soil texture, community rRNA, microbial diversity, indicator species, bioenergy, necromass

## Abstract

Bioenergy crops are a promising energy alternative to fossil fuels. During bioenergy feedstock production, crop inputs shape the composition of soil microbial communities, which in turn influences nutrient cycling and plant productivity. In addition to cropping inputs, site characteristics (e.g., soil texture, climate) influence bacterial and fungal communities. We explored the response of soil microorganisms to bioenergy cropping system (switchgrass vs. maize) and site (sandy loam vs. silty loam) within two long-term experimental research stations. The live and total microbial community membership was investigated using 16S and ITS amplicon sequencing of soil RNA and DNA. For both nucleic acid types, we expected fungi and prokaryotes to be differentially impacted by crop and site due their dissimilar life strategies. We also expected live communities to be more strongly affected by site and crop than the total communities due to a sensitivity to recent stimuli. Instead, we found that prokaryotic and fungal community composition was primarily driven by site with a secondary crop effect, highlighting the importance of soil texture and fertility in shaping both communities. Specific highly abundant prokaryotic and fungal taxa within live communities were indicative of site and cropping systems, providing insight into treatment-specific, agriculturally relevant microbial taxa that were obscured within total community profiles. Within live prokaryote communities, predatory *Myxobacteria* spp. were largely indicative of silty and switchgrass communities. Within live fungal communities, *Glomeromycota* spp. were solely indicative of switchgrass soils, while a few very abundant *Mortierellomycota* spp. were indicative of silty soils. Site and cropping system had distinct effects on the live and total communities reflecting selection forces of plant inputs and environmental conditions over time. Comparisons between RNA and DNA communities uncovered live members obscured within the total community as well as members of the relic DNA pool. The associations between live communities and relic DNA are a product of the intimate relationship between the ephemeral responses of the live community and the accumulation of DNA within necromass that contributes to soil organic matter, and in turn shapes soil microbial dynamics.

## Introduction

Bioenergy crop production provides a promising opportunity to decrease energy dependence on fossil fuels and limit increases in atmospheric carbon dioxide (CO_2_) concentrations (Gelfand et al., 2013). Low-nitrogen, marginal lands are targeted for bioenergy feedstock production to reserve productive land for food crops while increasing soil organic matter (SOM) stocks (Paustian et al., 2016). SOM formation and nutrient cycling are mediated *via* decomposition of plant inputs by soil biota, including microorganisms. Microbial community composition influences nitrogen (N) availability and carbon (C) mineralization, while their biomass and biochemistry influence the production of microbial necromass and the formation of persistent SOM (Simpson et al., 2007; Kallenbach et al., 2016; Crowther et al., 2019; Liang et al., 2019). Therefore, understanding how bioenergy cropping systems influence microbial community composition and necromass production is fundamental to sustainable bioenergy crop production (Jansson and Hofmockel, 2020; Zhu et al., 2020; Kasanke et al., 2021).

Annual (e.g., maize, *Zea mays* L.) and perennial (e.g., switchgrass, *Panicum virgatum* L.) bioenergy crops recruit diverse microbial communities with varied agronomic benefits (Liang et al., 2012a; Hargreaves et al., 2015; Jesus et al., 2016). Microbial recruitment is influenced by crop inputs, such as plant litter and root exudation, with annuals and perennials differing in their spatial and temporal delivery of these inputs (Hargreaves and Hofmockel, 2014). After the majority of aboveground biomass is harvested for bioenergy production, live and dead roots are the dominant source of new substrates supporting soil microbial decomposition (Sanford et al., 2016). In addition, perennial crops, like switchgrass, have year-round live root systems which can recruit symbiotic, nutrient-acquiring microbes such as arbuscular mycorrhizal fungi (AMF; Mafa-Attoye et al., 2020). The extent of AMF recruitment varies with sampling time and plant type with higher AMF abundance seen early in the growing season (Hijri et al., 2006) and after long-term (>10year) perennial management (Jesus et al., 2016). Perennials also have more extensive root systems than annuals, which have been shown to sustain a more diverse, but not necessarily larger (μg biomass C g^−1^ soil) microbial community (Liang et al., 2012a; Dou et al., 2013; Hargreaves and Hofmockel, 2014). Cropping system also incorporates the different fertilization requirements of each plant type, which is generally higher for annual plants than for perennials. A higher fertilization rate can impact microbial recruitment by decreasing the necessity for plants to support nutrient-acquiring symbionts such as AMF, and thereby lowering AMF root colonization and/or diversity (Oates et al., 2016; Emery et al., 2017; Jach-Smith and Jackson, 2018). However, other studies show no or a positive AMF response to fertilization (Treseder and Allen, 2002; Egerton-Warburton et al., 2007; Cheng et al., 2013). It is essential to understand the plant-microbe interactions within bioenergy cropping systems, to optimize microbial contributions to C and nutrient cycling, plant productivity, and long-term soil C storage.

In addition to the influences of cropping system on microbial community composition, variability in soil characteristics between experimental sites is often the greatest determinant in explaining soil microbial community differences (Mao et al., 2013; He et al., 2017; Zhou et al., 2017; Xue et al., 2018). Soil properties that influence soil moisture and nutrient status, such as texture, fertility, and pH, have been correlated to changes in bacterial and fungal community composition within unmanaged systems (Lauber et al., 2008; Vieira et al., 2019). Specifically, bacterial communities are shown to be structured by soil type and pH due to their sensitivity to microhabitat conditions as well as nutrient status (Ranjard and Richaume, 2001; Girvan et al., 2003; Fierer and Jackson, 2006; Fierer et al., 2009; Ramirez et al., 2012). Fungal communities are shown to be influenced by differences in soil moisture and nutrient status (Lauber et al., 2008; Talbot et al., 2014; Peay et al., 2016). Studies on agroecosystem soil microbiomes have largely focused on the effect of management type (e.g., land-use change, tillage, and biochar; Zhang et al., 2016; Sheng and Zhu, 2018; Gu et al., 2019); yet the contemporary and cumulative effects of bioenergy crops on the soil microbiome is still lacking, especially in a long-term (decadal), cross-site context (Liang et al., 2012b; Jesus et al., 2016).

Although the importance of cropping system and site characteristics in defining the assembly and function of microbial communities is generally recognized, there are still gaps in our knowledge of how these factors differentially impact microbial community membership to enhance necromass production and SOM formation. This is because the soil microbiome is extremely diverse and the community structure varies over time and space, making generalizable patterns difficult to identify. The influence of contemporary environmental conditions on microbial community structure may be best characterized with RNA-based measures, while DNA provides an integrated signature of both past and present microbiomes (Girvan et al., 2003; Orellana et al., 2019). Though there remains uncertainty surrounding the interpretation of RNA and DNA measurements, current consensus suggests that RNA-inferred communities include both metabolically active and dormant cells; therefore, RNA-based analyses are thought to represent organisms that may quickly respond to changes in environmental conditions (Anderson and Parkin, 2007; Blazewicz et al., 2013; Emerson et al., 2017). DNA is more stable in the environment; therefore, it is a less sensitive measure of temporal shifts in environmental microbiomes. Like RNA communities, DNA communities include active and inactive live cells. In addition, DNA communities include the roughly 40% of bacterial and fungal DNA that originates from non-living cells (“relic” DNA), whose persistence is a result of factors such as pH and soil mineralogy (Carini et al., 2016; Lennon et al., 2018). This relic DNA can persist for years and obscure measures of the dominant, live members, potentially skewing interpretations of microbial contributions to ecosystem function (Nagler et al., 2018). At the same time, this persistent relic DNA can potentially help identify organisms that produce necromass and contribute to SOM formation. While we recognize that RNA and DNA-inferred community interpretation is a dynamic field, we will refer to RNA and DNA communities from this point on as “live” and “total,” respectively. Past literature has found markedly different dominant members in live and total communities during SOM decomposition (Baldrian et al., 2012), after long-term N deposition (Freedman et al., 2015), following forest to plantation land conversion (Meyer et al., 2019), and after pulses of OM and metalloids (Birrer et al., 2018). Live bacterial communities have also been shown to be less diverse than total bacterial communities in bulk and rhizosphere rice paddy soils (Li et al., 2019). Assessing differences in live and total community diversity is a powerful approach for characterizing the response of microbial communities to contemporary disturbances as well as long-term environmental change.

The contributions of both the live and total communities are critical to developing cropping systems that manage microbiomes to promote plant production and enhance SOM formation. To date, live and total microbial community analyses have not been compared within bioenergy cropping systems which is important for understanding how promising bioenergy crops, like switchgrass, alter microbial community composition and microbially-driven processes. By characterizing live and total microbial communities at long-term maize and switchgrass sites with differing soil types, we have the unique opportunity to address this knowledge gap. This brings us to our main question: How do crop and site differentially influence the live and total prokaryotic and fungal communities within bioenergy cropping systems? H1: Fungal community composition is primarily influenced by crop type (perennial vs. annual) due to the importance of symbiotic relationships between plants and fungi for fungal metabolism. H2: Prokaryotic community composition is primarily influenced by soil type (sandy vs. silty) due to the importance of microhabitats created by soil texture. H3: Live prokaryotic and fungal community compositions are more sensitive to crop and site than their total community counterparts because they represent the most recent field stimuli without the influence of relic DNA.

## Materials and Methods

### Experimental Site and Design

Switchgrass and maize plots are located at the W.K. Kellogg Biological Station (KBS) Long-Term Ecological Research Site (Hickory Corners, MI, 42°24'N, 85°24'W) and at the Arlington Agricultural Research Station (AARS) of the University of Wisconsin–Madison (Arlington, WI, 43°18’N, 89°20’W) and as part of the Great Lakes Bioenergy Research Center (GLBRC) Intensive Biofuel Cropping System Experiments. AARS and KBS have similar climates, but different soil textures as outlined in detail within Sanford et al., (2016). Between 2008 and 2017, the KBS site received an average precipitation of 998mmyr.^−1^ and temperature of 9.3°C, while the AARS site received 904mmyear^−1^ and 7.0°C. Soil texture varies between the sites from a sandy loam (Mesic Typic Hapludalf) at the KBS site and a silty loam (Mesic Typic Argiudoll) at the AARS site (Soil Survey Staff, 2019). The sandy loam has a lower percent total soil C and N than the silty loam (~1% C, 0.1% N compared to 2% C, 0.2% N). Both sites have an average pH of 6.3 (Kasanke et al., 2021).

Switchgrass and maize plots were established at both the sandy and silty loam sites in 2008 and replicated in five blocks at each site. Details on the larger experimental design can be found within Sanford et al., (2016). Both sites practice no-till and receive fertilization rates based on an annual spring soil test to determine N, P, and K needs for optimal crop production: roughly 168kgNha^−1^ year^−1^ for maize and 56kgNha^−1^ year^−1^ for switchgrass. Aboveground biomass is harvested for biofuel production, leaving approximately 10cm stubble in maize plots, and 15cm stubble in switchgrass plots to decompose in place (Sanford et al., 2016). Maize plots had urea-ammonium fertilizer applied while planting (May 5, 2017 AARS; May 10, 2017 KBS). In addition, potash was applied to KBS maize plots on May 1, 2017. KBS switchgrass plots were fertilized May 10, 2017 and AARS switchgrass plots were fertilized with urea-ammonium fertilizer on June 2, 2017.

### Soil Sampling

Soils were collected from five switchgrass and five maize blocks within a larger experimental design at AARS on May 22, 2017 and from KBS on May 25, 2017. To test the effects of site and cropping system on the live and total microbial communities, samples were collected early in the growing season prior to plant emergence when plant inputs and rhizodeposition are at a minimum. This timing enabled us to test if RNA measurements reflect a community adapted to a cropping system or site and if it responds to current environmental conditions of increasing spring temperatures and soil moisture. For fertilized sites (all but AARS switchgrass), we also anticipate a strong signal from N cycling organisms. DNA measurements from this sampling time are aimed to measure the cumulative community within a site and cropping system over the past decade, including a strong necromass signature that may be difficult to differentiate when plant inputs are stimulating a greater diversity of the belowground community. To avoid sampling rhizosphere soil, cores were taken away from previous years’ stalks and obvious roots were discarded prior to soil analysis. Cores were collected using a 5-cm diameter hammer core to a depth of 15cm, and the upper 5cm was discarded to reduce the influence of litter. Replicate cores were taken from three locations within each plot and composited. Subsamples for nucleic acids were snap frozen in the field, shipped overnight on dry ice, and stored at −80°C prior to extraction.

### Environmental Variables

Salt-extractable organic C (DOC), and N (TDN), and microbial biomass C and N (MBC and MBN) were extracted from soil using a sequential chloroform fumigation direct extraction protocol (Witt et al., 2000; Hofmockel et al., 2007). Briefly, salt-extractable DOC/N was removed with 0.5M K_2_SO_4_ and the extract was filtered through a pre-leached Whatman ashless grade 42 filter. Microbial C and N were released by adding CHCl_3_ directly to the remaining soil sample before re-extracting in 0.5M K_2_SO_4_ to yield MBC/N. Non-purgeable organic C and total N were measured in acidified extracts on a Vario TOC cube (Elementar, Germany). Microbial biomass N and salt-extractable N were log-transformed to meet normality.

### RNA and DNA Extraction and Sequencing

Nucleic acids were co-extracted from 5g of soil using the Qiagen RNeasy PowerSoil Total RNA and RNeasy PowerSoil DNA Elution Kit (Qiagen, Hilden, Germany). DNA was purified with the Zymo DNA Clean & Concentrator Kit (Zymo Research, Irvine, CA, United States). DNA-based community measures all microbial cells, including relic DNA. RNA was treated with DNase (TURBO DNA-free, Invitrogen, Carlsbad, CA, United States) and quality was assessed using the RNA 6000 Nano Kit on an Agilent 2100 Bioanalyzer (Agilent Technologies, Santa Clara, CA, United States). cDNA was generated from 100ng of total RNA with the NEBNext RNA First Strand Synthesis Module, and NEBNext Ultra II Non-Directional RNA Second Strand Synthesis Module (New England Biolabs, Ipswich, MA, United States), before cleanup with the Zymo DNA Clean & Concentrator Kit. The RNA community consists of active and dormant cells while DNA encompasses the RNA community with the addition of relic DNA (DeAngelis and Firestone, 2012; Blagodatskaya and Kuzyakov, 2013; Blazewicz et al., 2013).

Sequencing was performed at The Environmental Sample Preparation and Sequencing Facility at Argonne National Laboratory (Lemont, IL, United States). Samples were prepared according to standard Earth Microbiome Project amplicon sequencing protocols (Caporaso et al., 2011). Briefly, we used high throughput sequencing of the V4 variable region of the16S rRNA gene with 515F (Parada et al., 2016)–806R (Apprill et al., 2015) forward barcoded primers for bacterial/archaeal analysis, and the ITS region with ITS1f- ITS2 (White et al., 1990; Smith and Peay, 2014) for fungi. Sequencing rRNA from the ITS region captures actively transcribing fungi because ITS regions are removed during post-transcriptional modification (Coleman, 2015). Post-transcriptional modification does not occur in prokaryotes, thus 16S rRNA sequencing also includes live, but dormant cells and spores (Blazewicz et al., 2013). Thus, for simplicity, we refer to the communities identified *via* rRNA sequencing as “live” and the community identified by rRNA gene sequencing as the “total” communities, which includes relic DNA (Carini et al., 2016). Libraries were sequenced on an Illumina MiSeq (Illumina, San Diego, CA, United States) using paired end 151×151 (16S) or 250×250 (ITS) chemistry (Caporaso et al., 2011).

### Bioinformatics

Quality control, merging, and database selection were performed with Hundo (Brown et al., 2018). BBDuk2 of the BBTools package was used to quality trim paired-end sequences and remove contaminant sequences (Brown et al., 2018). VSEARCH merged passing reads and aggregated them into a single FASTA file. OTU sequences were aligned to a reference database using BLAST. 16S sequences were referenced against the SilvaMod database curated by the CREST team from SILVA nr SSU Ref v128, and ITS against UNITE v7.2 using the CREST LCA Classifier (Lanzén et al., 2012). OTU sequences were filtered if they contained fewer than three sample reads. Taxa were removed if they were not bacterial/archaeal (16S) or fungal (ITS). Normalization method was examined by comparing the diversity results of rarefication, which is a stringent normalization technique that discards a large proportion of sequencing reads and omits some rare OTUs, and cumulative-sum scaling (CSS) which corrects for differences in sampling depth while keeping total count variations between samples, and retains all OTUs (Paulson et al., 2013). Samples were normalized two different ways: rarifying each sample to 21,000 reads or cumulative-sum scaling (CSS) to understand if normalization method affected diversity metrics. Diversity and evenness metrics were compared between rarified and normalized data prior to running statistical analyses. Diversity and evenness were similar between rarified and normalized data with the exception of live and total fungal evenness (Supplementary Table 1); therefore, to maximize data retention, CSS-normalized values were used for subsequent analyses. Full OTU tables for each community and organism are included in Supplementary File 1.

### Statistical Analyses

Crop (switchgrass and maize) and site (sandy or silty loam) effects on microbial community composition were tested for RNA and DNA fungal and prokaryotic communities using packages and workflow within the R software package pmartRseq (R Core Team, 2018). Differences in relative abundance percentage between RNA and DNA communities were tested using a mixed model ANOVA with block as a random factor (*α*=0.05) followed by pairwise comparisons using Tukey’s HSD within the emmeans package (Lenth et al., 2020). Within-sample diversity and was evaluated by alpha diversity using Inverse Simpson’s index. The distribution of abundance across OTUs was evaluated using Pielou’s evenness index. The effect of crop and site on bacterial and fungal alpha diversity measures was assessed using a mixed model ANOVA with block as a random factor (*α*=0.05). The compositional difference between samples was measured by beta diversity *via* Bray-Curtis distance matrices as a function of crop and site, using permutational multivariate analysis of variance (PERMANOVA) within the vegan package (Oksanen et al., 2019). Pairwise comparisons were calculated after the value of *p* adjustment using Tukey’s HSD within the emmeans package (Lenth et al., 2020). Indicator species analyses were employed to test for microbial species that consistently associate with crop and site treatments. Indicator species were identified as significant at *α*<0.05 and indicator value (IV)>0.7 for a specific crop or site, using the indicspecies package and sorted by mean abundance to compare the top species (De Cáceres and Legendre, 2020). Briefly, a strong indicator species is found largely and solely within a specific group. A higher number of indicator species for a site or crop indicates the presence of a unique community fostered by site or crop characteristics. The higher the strength of the indicator value, the higher the likelihood that the species will only appear within certain sites or crops.

## Results

### Live and Total Fungal Communities

**Figure 1:**
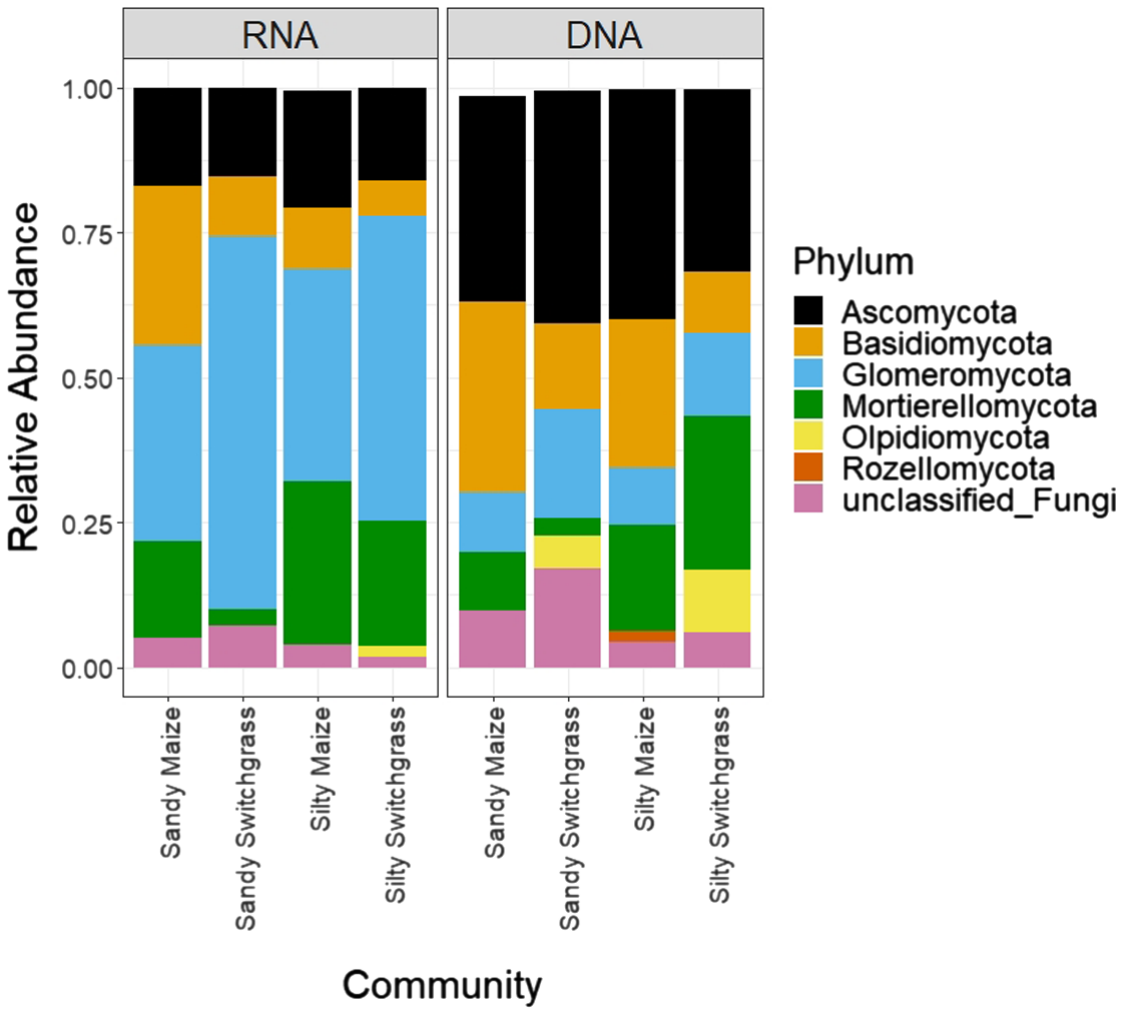
Relative normalized abundance of fungal phyla separated by site/crop combination based on sequencing of ITS rRNA gene fragments of RNA and DNA (*n*=5).

After CSS normalization and removal of low counts, there were 2,258 fungal OTUs with 997 OTUs found within the live (RNA) community and 2,048 OTUs found within the total (DNA) community. The dominant membership between the live and total communities were different as indicated by little overlap between the top five most abundant OTUs for each site, crop, and site/crop combination (Supplementary Tables 2 and 3). Figure 1 and Table 1 revealed stark differences between live and total community composition with a significantly higher proportion of Glomeromycota (+32.6%) in the live community and Ascomycota (+19.7%) in the total community, thus the live and total communities were subsequently analyzed separately due to their distinct community compositions.

**Table 1:** Average and standard error (in parentheses) of CSS-normalized relative abundance percentage within communities or sites/crops for fungal (ITS) and bacterial (16S) communities (RNA or DNA *n*=20; site or crop *n*=10).

	Taxonomy	RNA	DNA	RNA				DNA			
				Sandy	Silty	Maize	Switchgrass	Sandy	Silty	Maize	Switchgrass
ITS	Ascomycota	17.4 (1.4)	37.1 (1.4)*	-	-	-	-	-	-	-	-
	*Pleosporales*	4.5 (1.0)	8.0 (0.9)*	-	-	-	-	-	-	-	-
	Basidiomycota	14.0 (2.8)	**21.5 (2.7)**	**19.0 (5.0)**	9.0 (1.6)	**19.0 (4.5)**	9.0 (2.8)	-	-	29.2 (3.5)*	13.8 (2.4)
	*Agaricales*	4.7 (1.6)	**7.3 (2.1)**	**6.2 (2.9)**	3.2 (1.3)	**6.7 (3.0)**	2.6 (1.1)	-	-	10.8 (3.6)*	3.7 (1.5)
	Glomeromycota	47.2 (4.0)*	13.7 (1.7)	-	-	35.3 (4.6)	59.1 (3.7)*	-	-	10.2 (2.3)	17.2 (1.9)*
	*Glomerales*	34.2 (3.5)*	11.5 (1.5)	-	-	25.6 (3.6)	42.7 (4.8)*	-	-	8.9 (1.9)	14.0 (2.0)*
	Mortierellomycota	**16.4 (2.9)**	13.5 (3.2)	9.7 (3.3)	23.2 (4.0)*	22.4 (4.2)*	10.5 (3.4)	6.6 (1.6)	20.4 (5.5)*	-	-
	*Mortierellales*	**16.3 (3.0)**	13.3 (3.2)	9.5 (3.3)	23.2 (4.0)*	22.4 (4.2)*	10.3 (3.4)	6.4 (1.6)	20.1 (5.5)*	-	-
	Olpidiomycota	-	-	-	-	-	-	-	-	0.4 (0.2)	**7.3 (4.0)**
	*Olpidiales*	-	-	-	-	-	-	-	-	0.4 (0.2)	**7.3 (4.0)**
16S	Acidobacteria	17.8 (0.8)	23.1 (0.8)*	20.8 (0.7)*	14.9 (0.7)	-	-	25.9 (0.5)*	20.4 (0.8)	-	-
	*Blastocatellales*	1.9 (0.1)	8.7 (0.6)*	-	-	-	-	10.6 (0.6)*	6.9 (0.6)	-	-
	*Solibacterales*	-	-	6.5 (0.5)*	3.6 (0.4)	-	-	-	-	-	-
	Proteobacteria	39.5 (1.0)*	21.0 (0.8)	36.9 (0.6)	42.1 (1.4)*	-	-	-	-	-	-
	*Myxococcales*	20.6 (1.1)*	3.6 (0.3)	16.3 (0.7)	24.9 (0.9)*	-	-	-	-	-	-
	Verrucomicrobia	8.7 (0.3)	18.5 (0.9)*	-	-	-	-	-	-	-	-
	*Chthoniobacterales*	1.1 (0.0)	11.3 (0.9)*	-	-	-	-	-	-	-	-

Phyla shown exhibited 5% or greater difference in relative abundance between RNA and DNA communities as well as the order (shown in italics) exhibiting the greatest difference in relative abundance. The higher relative abundance between communities is bolded. *indicates significance at *p*<0.05.

### Site and Cropping Influences on Fungal Community Composition

**Figure 2:**
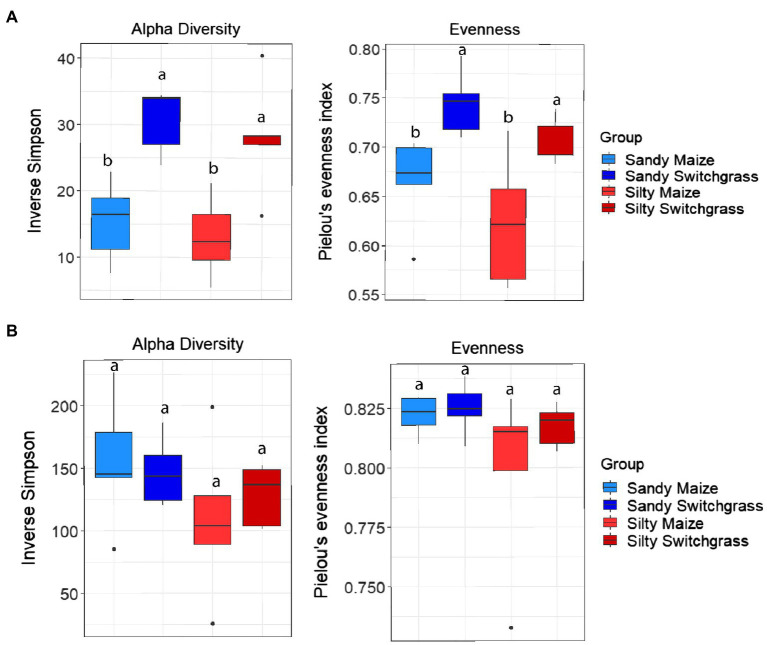
Alpha diversity and evenness comparing site and crop influences on RNA **(A)** and DNA **(B)** fungal communities. Boxes encompass the 25th to 75th percentiles and whiskers extend to the maximum and minimum except when a point exceeds 150% of the interquartile range. Black bars indicate the mean and black dots denote outliers. Letters denote significance at *p*<0.05.

Further analysis revealed that crop, site, and their interaction influenced fungal community composition in both the live and total communities (PERMANOVA, *p*<0.05; Table 2). Despite distinct membership (Figure 1), both communities exhibited a similar site by crop interaction (*R*^2^=0.07). However, site was slightly more influential in driving community compositions (RNA *R*^2^=0.14; DNA *R*^2^=0.20) than cropping system (RNA *R*^2^=0.09; DNA *R*^2^=0.13). The live switchgrass community had higher diversity (inverse Simpson, *p*=0.001, 29.32±6.72 vs. 14.15±5.88) and a more even species distribution (Pielou’s, *p*=0.001, 0.73±0.03 vs. 0.64±0.06) than maize (Figure 2). Total communities did not exhibit differences in diversity or evenness between cropping system or site (Figure 2). In the live community, silty soil had a significantly higher proportion of Mortierellomycota (+13.5%) while sandy soil contained more Basidiomycota (+9.9%). Live maize communities had a higher proportion of Basidiomycota (+10.0%) and significantly more Mortierellomycota (+11.9%) compared to the switchgrass live community, which contained significantly more Glomeromycota (+23.9%; Table 1). In the total community, silty soil had a significantly higher proportion of Mortierellomycota (+13.8%) than sandy soil. Total maize communities had a greater proportion of Basidiomycota (+15.3%) than the switchgrass total community, which had significantly more Glomeromycota (+7.0%) and marginally more Olpidiomycota (+6.9%; Table 1).

**Table 2:** Results from permutational multivariate analysis of variance (PERMANOVA) statistical tests showing the effects of site, crop, and their interactions on Bray–Curtis distance matrices for both fungal and bacterial communities (*p≤0.05; **p≤0.01; ***p≤0.001).

Within Live Community											
**Fungi**	df	F	R^2^	p		**Bacteria**	df	F	R^2^	p	
Site	1	3.5596	0.1525	0.001	***	Site	1	9.2599	0.29223	0.001	***
Crop	1	2.2904	0.09812	0.001	***	Crop	1	4.1857	0.13209	0.002	**
Site*Crop	1	1.4924	0.06394	0.04	*	Site*Crop	1	2.2417	0.07074	0.043	*
Within Total Community											
**Fungi**	df	F	R^2^	p		**Bacteria**	df	F	R^2^	p	
Site	1	5.3805	0.19825	0.001	***	Site	1	7.7906	0.27021	0.001	***
Crop	1	3.4946	0.12876	0.001	***	Crop	1	2.9881	0.10364	0.003	**
Site*Crop	1	2.2646	0.08344	0.005	**	Site*Crop	1	2.053	0.07121	0.024	*

### Fungal Site and Crop Indicator Species

**Figure 3:**
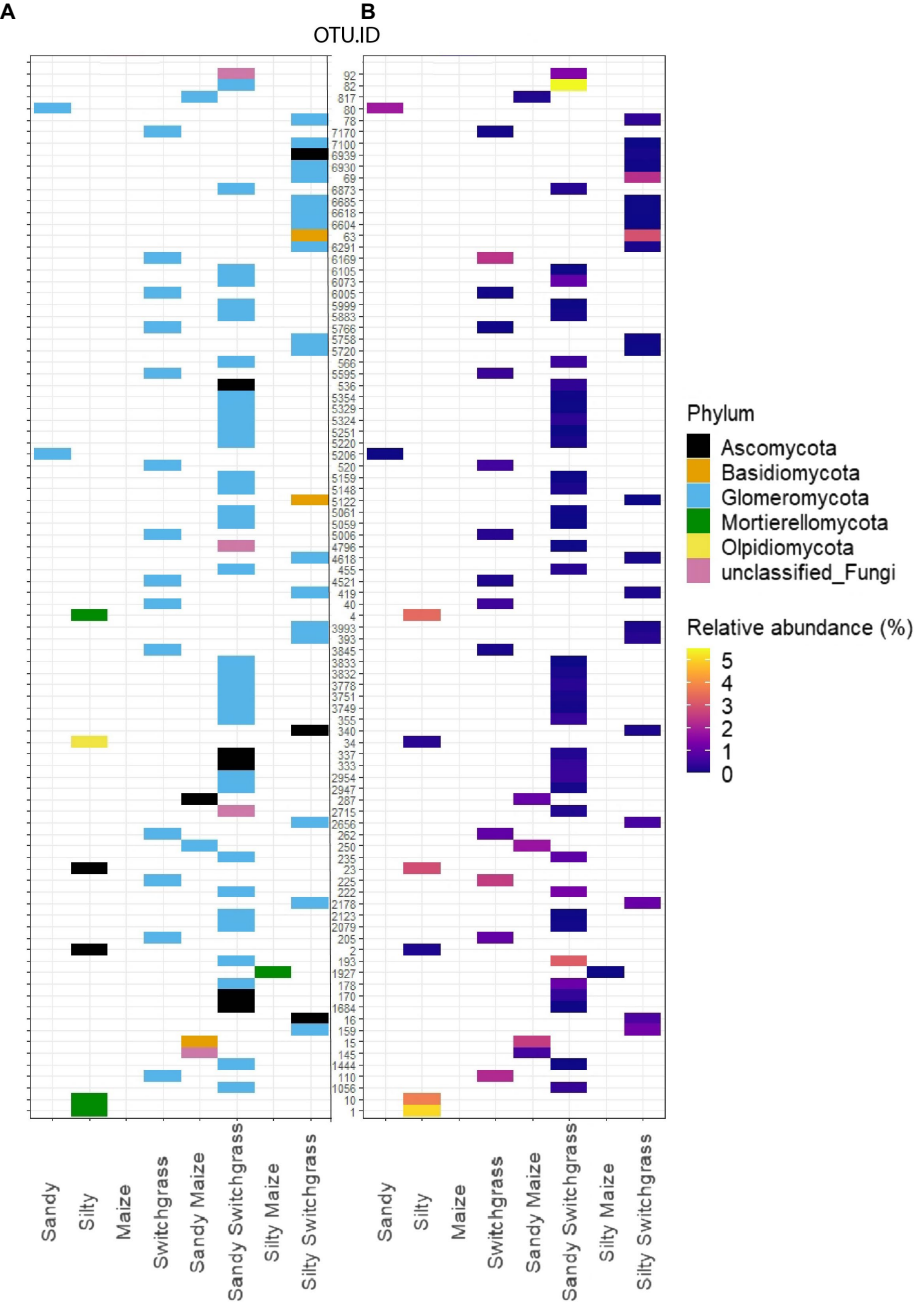
Schematic representation of all RNA fungal indicator species within each site, crop, and site/crop combination which can be found within Supplementary File 2. Indicator species represented as a heatmap matrix colored by phylum **(A)** and average relative abundance (%; **B**). OTU were selected by significance (*α*<0.05) and indicator value (IV>0.7) for a specific crop or site.

Indicator species were filtered on indicator value strength (IV>0.7) and significance (*α*<0.05) for sites, crops, and crop/site combinations (Supplementary Tables 4 and 5; Supplementary Files 2 and 3). The most distinct taxonomic differences were observed for site or crop indicator species rather than within site/crop combinations (Figure 3; Supplementary Figure 1). Within the live community, switchgrass communities had 14 indicator species while no indicator species were detected for maize (Supplementary File 2). All switchgrass indicator species were Glomeromycota, of which the most abundant indicator OTU was the second most abundant fungal OTU in switchgrass plots (Supplementary Table 4). Within sites, silty soil had six indicator species while sandy soil had two. The top three silty soil indicator species in the live community were from the family Mortierellaceae and were highly abundant and loyal to silty communities with an average IV of 1.00 (Supplementary Table 4). Sandy and silty switchgrass indicator species were largely from Glomeromycota as well (Figure 3). Within the total community, sandy sites had the most indicator species (93), followed by silty (65), switchgrass (59), and maize (35; Supplementary File 3). The total community (Supplementary Figure 1) had more taxonomic variety within its most abundant indicator species compared to the live community (Figure 3). Two of the top five total silty soil fungal indicator species were members of Mortierellomycota of which the most abundant was the most abundant silty OTU. Only one of the top five switchgrass total fungal indicator species was a member of Glomeromycota, and the most abundant species was *Hannaella pagnoccae* (3.1±5.5%; Supplementary Table 5). A similar result was found within crop/site combinations with a few sandy and silty switchgrass indicator species from Glomeromycota (Supplementary Figure 1).

### Live and Total Prokaryotic Community

**Figure 4:**
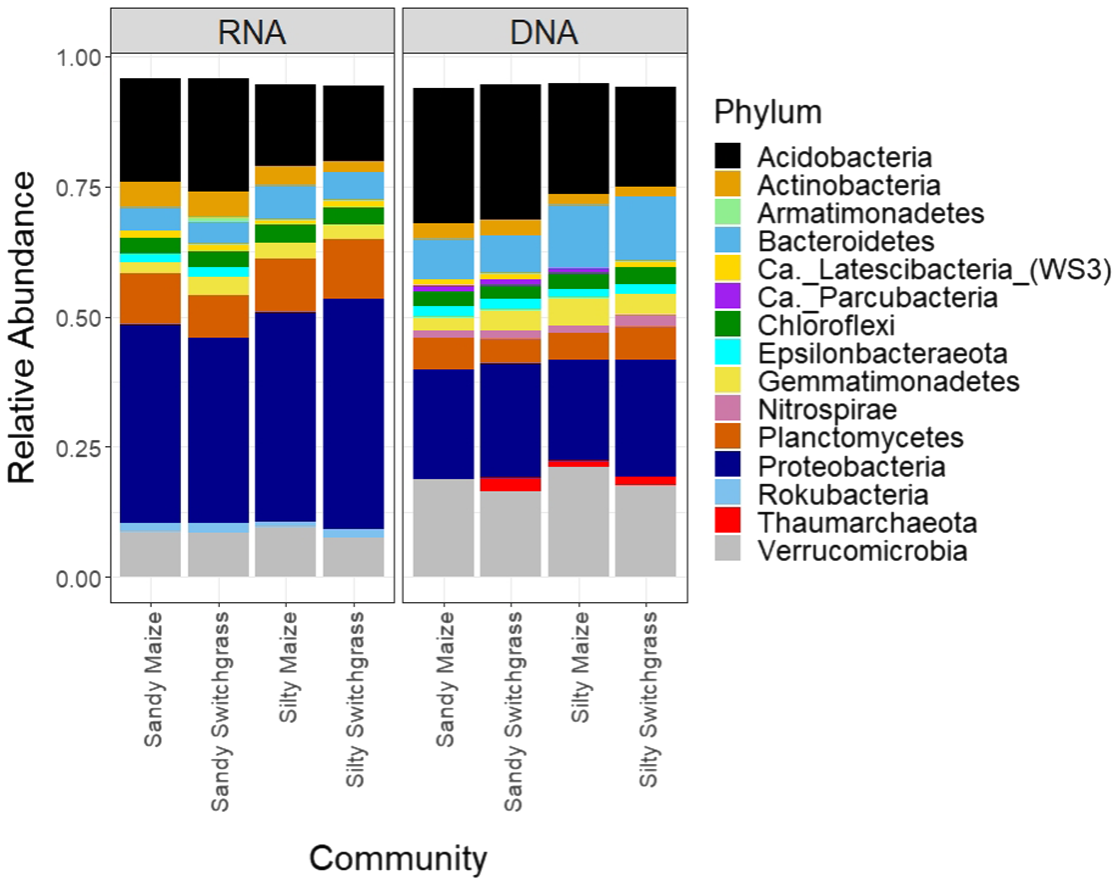
Relative abundance of bacterial and archaeal phyla separated by site/crop combination based on sequencing of 16S rRNA gene fragments at the DNA and RNA level (*n*=5).

After CSS normalization and removal of rare reads (<2), there were 13,095 prokaryotic OTUs with 9,962 OTUs found within the live community and 10,669 OTUs found within the total community. There were 106 archaeal OTUs out of the 12,900 total OTUs (0.8%), none of which were highly abundant (Supplementary Tables 6 and 7). From this point forward, the prokaryote community will be referred to as the bacterial community although a few archaea are included within the analyses. There was little overlap between live and total communities within the top five most abundant OTUs for each site, crop, and combination (Supplementary Tables 6 and 7). The live community was taxonomically distinct from the total with a significantly higher proportion of Proteobacteria (+18.9%) while the total community had a higher proportion of Acidobacteria (+5.5%) and Verrucomicrobia (+10.0%; Figure 4; Table 1).

### Site and Cropping Influences on Bacterial Community Composition

**Figure 5:**
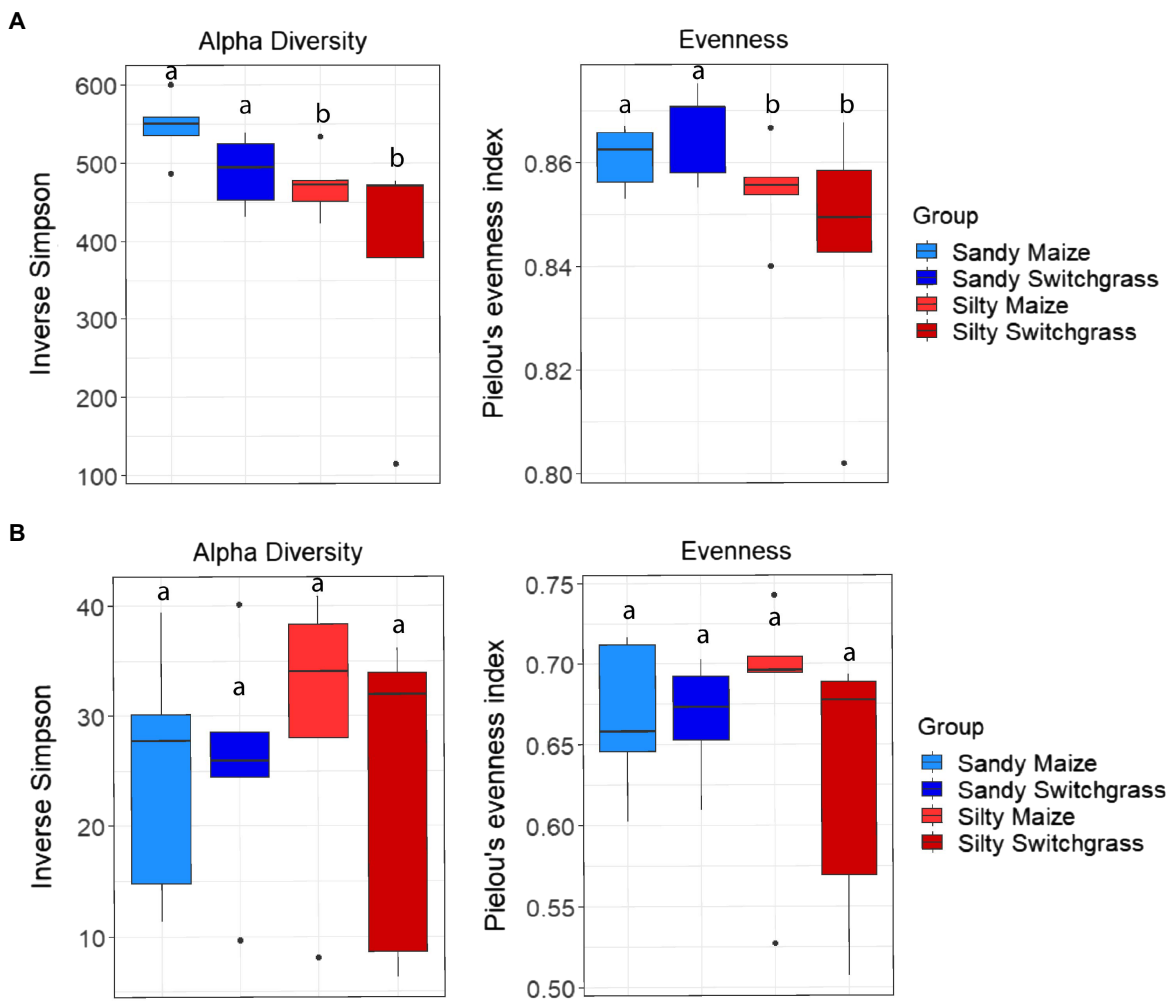
Alpha diversity and evenness comparing site and crop influences on the RNA **(A)** and DNA **(B)** bacterial communities. Boxes encompass the 25th to 75th percentiles and whiskers extend to the maximum and minimum except when a point exceeds 150% of the interquartile range. Black bars indicate the mean and black dots denote outliers. Letters denote significance at *p*<0.05.

Like our fungal analyses, live and total communities were analyzed separately to distinguish communities with and without relic DNA. Site, crop, and their interaction all had a significant influence on bacterial community composition in both the live and total communities (PERMANOVA, *p*<0.05). It is important to note that treatment effects explained more of the variation in community composition within bacterial communities compared to fungal communities. Despite different community compositions, live and total bacterial communities exhibited a similar site by crop interaction (*R*^2^=0.07). They also had similar crop and site effects with site as the primary driver within both communities (RNA *R*^2^=0.29; DNA *R*^2^=0.27) and cropping system secondary (RNA *R*^2^=0.13; DNA *R*^2^=0.10). In both nucleic acid types, site influenced differences in alpha diversity, while cropping system did not. The live sandy soil community had a more even species distribution (Pielou’s, *p*=0.045, 0.86±0.01) than silty soil (0.85±0.02), while the total sandy community was more diverse (inverse Simpson, *p*=0.032, 517.14±51.21) than silty soil (426.61±116.71; Figure 5). In the live community, sandy soil had a significantly higher proportion of Acidobacteria (+5.9%) while silty soil had significantly more Proteobacteria (+5.2%). In the total community, sandy soil had a significantly higher proportion of Acidobacteria (+5.5%) than silty soil (Table 1).

### Bacterial Site and Crop Indicator Species

Indicator species were filtered for sites, crops, and crop by site combinations (Supplementary Tables 8 and 9; Supplementary Files 4 and 5). As expected, a much larger number of bacterial indicators than fungal indicators were found due to the higher diversity of the bacterial community overall, and the influence of micro-habitats on bacterial niches (Bach et al., 2018). Within the live community, silty communities had the most indicator species (740) compared to sandy (676), maize (276), and switchgrass (160) communities (Supplementary File 4). Indicator species taxonomic trends were not as distinct for bacteria as in fungal communities with top indicator species largely from Proteobacteria across all treatments. A substantial portion (28%) of live silty switchgrass indicator species were from the Proteobacteria order, *Myxococcales*. Additionally, the four most abundant indicator species within silty switchgrass were members of *Myxococcales* (Supplementary Table 8). Acidobacteria indicator species were concentrated within sandy and maize sites while Bacteroidetes and Planctomycetes indicators were in silty, silty/maize, or silty/switchgrass communities. One live archaeal species, the thaumarcheotal *Nitrososphaera viennensis*, was indicative of sandy/maize (Supplementary Figure 2). Within the total community, sandy communities had the most indicator species (598) compared to silty (518), maize (169), and switchgrass (119) communities (Supplementary File 5). Top indicator species were largely from Proteobacteria, but more Acidobacteria, Gemmatimonadetes, and Verrucomicrobia indicator species were present compared to the live community. The same archaeal indicator species found in the live community was indicative of sandy/maize in the total community (Supplementary Figure 3).

### Environmental Variables

We explored extractable and microbial C and N dynamics under maize and switchgrass at the sandy loam and silty loam sites. Mean values and standard deviations are included within Supplementary Table 10. Sandy and silty soil had similar C concentrations, but silty soil had significantly higher TDN (*p*<0.001) and marginally higher MBN concentrations (*p*=0.08). DOC:TDN ratios were highest for sandy soils (5.96±1.65 sandy; 2.62±0.53 silty) and sandy switchgrass (6.84±0.99). MBC:MBN ratios were highest for sandy soils (8.95±1.16 sandy; 5.12±1.40 silty).

## Discussion

### Despite Compositional Differences, Live and Total Communities Have Similar Environmental Drivers

We performed the first analysis of the influence of bioenergy cropping systems on live and total soil microbial communities to better understand the contemporary and lasting selective forces of cropping system and site selection on bacterial and fungal communities. We hypothesized that live communities would be more sensitive to contemporary conditions because they represent the most recent field stimuli without the influence of relic DNA (H3). Instead, we found that site and cropping system contributed similarly to selecting live and total community structure, although their community membership varied strongly (Tables 1 and 2). Our results differ from other studies that found a stronger treatment response in the live compared to the total community after addition of biochar to rice paddies (Chen et al., 2016) and influx of methane into a landfill biocover (Kim et al., 2013). A distinguishing factor of our study is the consistent field management during the previous 9years, while the cited studies measured the effect of acute treatments on the live community. Our results suggest that our consistent cropping system produced a strong present-day and legacy effect on microbial communities, validating the importance of DNA-based analyses for detecting lasting environmental impacts while highlighting the importance of RNA for identifying community members that are responsive to current environmental conditions (Zhang et al., 2014; Nawaz et al., 2019; Orellana et al., 2019; Wutkowska et al., 2019).

The cumulative effects of site and crop on community structure explained 18% more variation within live bacterial communities than for live fungal communities (0.49 vs. 0.31; Table 2). These findings indicate that bacteria and fungi react differently to biotic and abiotic factors, and we are missing a larger portion of the variation behind fungal community composition when only accounting for broad effects such as cropping system and site. Site and cropping system explained a similar amount of variance to past studies at our experimental sites (Jesus et al., 2016), yet a large portion was left unexplained likely due to several unaccounted drivers such as phosphate concentration and soil moisture (Fierer et al., 2009; Kuramae et al., 2012; Deepika and Kothamasi, 2015). Stochastic processes, such as random birth/death events, are inherently unpredictable and are likely playing a role in the unexplained variance as well (Evans et al., 2017).

### Site Was the Primary Influence on Bacterial and Fungal Communities

While we were supported in hypothesizing that site would be the primary driver of prokaryotic community composition (H2), we did not anticipate a primary site effect within the fungal community as well. Even though live and total communities differed in composition, they were similarly influenced by environmental factors (Table 2), which has been shown within forested and aquatic systems (Romanowicz et al., 2016; Nawaz et al., 2019). Site can encompass many attributes; because pH and climate are similar between our two study sites, soil type and nutrient status are likely the main drivers of community structure. The importance of texture and nutrient status indicates a reliance on habitat niche, a pattern consistent with previous studies (Girvan et al., 2003; Oehl et al., 2010; Jesus et al., 2016; Jach-Smith and Jackson, 2018; Kasanke et al., 2021). It is interesting to note that Jesus et al. (2016) studied the same field sites and found a strong site effect on DNA communities after 2years of establishment (Kasanke et al., 2021), and after 8years of establishment also found a strong long-term site effect. The novelty of our results come from the inclusion of RNA-based data as well as sampling pre-growing season, which reveal that the strong site effect on microbial community characteristics is consistent for both the time-integrated total community and the contemporary live community. DNA is appropriate for some questions, particularly when comparing communities across time or space, but is less ideal for deciphering relationships between environmental effects and microbial members due to differences in dominant membership between the live and total community.

### Crop Had a Secondary Influence on Bacterial and Fungal Communities

We hypothesized that crop type would have a stronger impact than site in shaping fungal communities due to the importance of plant inputs as a carbon/energy source for fungal metabolism (H1), but live and total communities sampled pre-growing season did not support this hypothesis. Crop management explained a smaller but significant amount of variance in both bacterial and fungal communities, yet the live and total communities were composed of different proportions of members (Tables 1 and 2). Both communities uncovered the prevalence of Basidiomycota in maize plots and Glomeromycota under switchgrass. Live and total communities revealed different dominant crop-specific members with a higher proportion of Mortierellomycota within live maize communities and more Olpidiomycota found within total switchgrass communities (Table 1). Mortierellomycota contains several genera of saprotrophic fungi which points to a possible prevalence of fungi decomposing recalcitrant maize residue left over from the previous season (Webster and Weber, 2007). Olpidiomycota is a new phylum composed primarily of plant-pathogenic genera which indicates a potential relationship between pathogens and perennial crops (Naranjo-Ortiz and Gabaldón, 2019). Even in the absence of actively growing plants, cropping system had a lasting impact on both the live and total community membership.

Although our conventional cropping system did not allow for a true test of crop type vs. fertilizer rate (i.e., lower N rates on maize and vice versa), it is a powerful systems approach which provides insight into long-term impacts of cropping management on the soil microbial community. Past studies have highlighted bioenergy crop type as an important secondary driver of bacterial community structure (Zhang et al., 2017), yet our results uncovered the universality of this trend (i.e., for fungi and within both live and total communities). Seasonality can affect the delivery of plant-derived substrates (i.e., aboveground litter production and root exudate production) as well as delivery of inorganic nutrients (i.e., fertilization). By sampling early in the growing season, rhizodeposition was minimized, thereby focusing the attention on the recent and long-term stimuli of fertilization and perennial/annual cropping, respectively. Other studies have documented the effect of rhizodeposition during the growing season (Upton et al., 2019; Wattenburger et al., 2019); we captured the persisting effects of cropping system and site by sampling pre-growing season. Jesus et al. (2016) found crop to be a greater driver than site within long-term (10years) bioenergy cropping systems which contradicts our primary site effect within both contemporary (RNA) and long-term (DNA) communities. This difference could be explained by differences in sampling time, wherein our pre-growing season soil conditions determined community composition instead of growing season crop inputs. While we expected recent N fertilization to heavily impact live communities, soil properties such as C/N are important for satisfying microbial metabolic needs (Zhao et al., 2019) and differed between sites (Supplementary Table 10) Although the crop effect was secondary, it was still significant within live communities, which indicates the influence of recent field management such as fertilization and crop types (perennial/annual).

### Myxobacteria Were Indicative of Silty Switchgrass Communities

Assessing which microbes are indicative of different soil niches and crops can assist in connecting the micron-scale functions of microorganisms to the broader ecological context (Bach et al., 2018; Biesgen et al., 2020). After identifying unique membership, relationships between membership and ecosystem processes were explored. Indicator species analyses on the bacterial community revealed a highly diverse suite of prokaryotic indicator species (59 phyla) within both the live and total communities (Supplementary Files 4 and 5). Unlike the fungal indicator species, specific phyla or orders of prokaryotes were not solely indicative of a site or cropping system. While the proteobacterial order, *Myxococcales*, was found within all sites, crops, and combinations, live silty switchgrass communities had the highest proportion of *Myxococcales* as well as highly abundant members (Supplementary Figure 2; Supplementary File 4). Members of *Myxococcales* represent facultative predatory bacteria which hunt in packs to lyse cells and consume necromass (Muñoz-Dorado et al., 2016; Hungate et al., 2021). They have been shown to prey on gram-negative bacteria and assimilate necromass carbon (Hanajima et al., 2019). It is intriguing to consider what characteristics of the spring pre-growing season conditions within silty and switchgrass communities were ideal for *Myxococcales* to bloom. The pre-growing season is characterized by low plant-derived inputs which might be an indication of the ability of Myxobacteria to survive and thrive when food is scarce.

### Mortierellomycota Were Strong Indicator Species for Silty Soil

Mortierellomycota species were top indicators for both live and total fungi within silty soil. Compared to sandy soils, the silty soil had higher total dissolved nitrogen (TDN) levels which were positively correlated with the abundance of genus Mortierella fungi at our sites (*R*^2^=0.22, *p*=0.001; Supplementary Figure 4) and reported within past studies (Detheridge et al., 2016). In addition to Mortierellomycota, both live and total silty switchgrass indicator species were dominated by AMF. The prevalence of Mortierella species might be beneficial to crop growth, since these species have been found to increase plant phosphorus uptake when in the presence of AMF (Osorio and Habte, 2001). Thus, silty switchgrass communities may benefit from microbe-microbe interactions between AMF and Mortierellomycota. Additionally, Mortierellomycota are generally fast-growing fungal species that thrive on organic substrate additions in arable soils (Schlatter et al., 2017). Thus, the higher C and N status of silty soil could have stimulated the unique occurrence of highly abundant Mortierella species. The abundant Mortierellomycota indicator species were shared between the live and total fungal communities, suggesting they are persistent and may be all-time ecologically important members of the fungal community. These results support the notion that Mortierella are potentially important contributors to microbial necromass production and SOM formation (Fernandez and Kennedy, 2018; Li et al., 2018; Kasanke et al., 2021).

### Glomeromycota (AMF) Were Strong Indicator Species for Switchgrass

Switchgrass supported unique fungal members, as demonstrated by higher counts of indicator species compared to maize within both the live and total fungal community. Switchgrass indicator species were predominantly Glomeromycota, especially within the live community, alluding to the importance of perennial rhizomes for early season switchgrass growth symbionts (Somenahally et al., 2018). AMF form associations with ~75% of plant species and acquire nutrients which could be especially important on marginal lands with limited nutrient pools (Emery et al., 2018). In native prairies, switchgrass is known to be strongly reliant on AMF (Bingham and Biondini, 2009), but this is the first RNA-based documentation of AMF activity in early season switchgrass bioenergy cropping systems. Switchgrass receives a much lower fertilization rate compared to maize which might also promote higher AMF colonization, since mycorrhizal abundance has been shown to negatively correlate with N fertilization within agriculture and across biomes (Treseder, 2004; Emery et al., 2017; Jach-Smith and Jackson, 2018). Sandy switchgrass had more indicator species than silty switchgrass and a higher proportion of Glomeromycota species. A higher proportion of Glomeromycota indicators in sandy soil coincides with past findings that sand content positively correlated with higher colonization of AMF fungi (Zaller et al., 2011). Members of the AMF-genus Rhizophagus were also marginally negatively correlated with salt-extractable N concentrations (*R*^2^=0.13, *p*=0.065; Supplementary Figure 5); thus, a lower N level could have encouraged Glomeromycota colonization in sandy soils like past studies (Detheridge et al., 2016). The prevalence of AMF is exciting because of their role in supporting plant health by scavenging nutrients from outside the rhizosphere (Chen et al., 2018). Although cropping system was a secondary microbial filter compared to soil type, crop-specific colonization of beneficial fungi under switchgrass demonstrates the lasting influence of cropping system on the soil microbiome even before the growing season.

### Live and Total Communities Highlight Different Members

Live and total bacterial communities contained different dominant members, making it difficult to link relatively abundant bacterial taxa to their environmental roles or preferred habitats using only an amplicon DNA-based approach (Table 1). DNA-based investigations into soil bacterial community structure are less technically involved and therefore much more common than RNA-based studies (Knight et al., 2018). While temporally sampling the DNA community can help distinguish between seasonal or event-driven blooms, RNA is important for highlighting live members continually concealed in the DNA community by a potentially large relic DNA pool (Carini et al., 2020). However, it is not possible to determine if members of the live community are active, perhaps except for ITS RNA sequencing due to the presence of ribosomes in dormant cells (Blazewicz et al., 2013). In addition, slow growing microorganisms in this environment (e.g., oligotrophic) may be less represented in the live community compared to the total community due to lower ribosome cell content. Alternatively, differences in fungal and bacterial live and total communities may be driven by a difference in residence time between bacterial and fungal DNA. Fungal cell walls are composed of recalcitrant materials which decay slowly, favoring their accumulation in soil (Li et al., 2015; Starke et al., 2019). This suggests that differences between total and live communities could be used to identify relic DNA from recalcitrant fungal biomass and provide a signature for taxa contributing to microbially-derived SOM (“necromass”; Liang et al., 2019). The communities represented by RNA and DNA sequencing is still a topic of debate and differentiating the *in-situ* diversity of living microbial biomass from long-term persistent signatures remains an important frontier. By uncovering unique, dominant members within the RNA-based community, our results emphasize the importance of recognizing that the DNA-based community is an integrative measure of past and present communities. To avoid the significant biases imposed by relic environmental DNA, other approaches (e.g., RNA, qSIP, and culturing) should be used in tandem for linking microbial taxonomy to environmental factors.

## Conclusion

Our study highlights the dynamic nature of live and total microbial communities under major bioenergy cropping systems (switchgrass and maize) at two sites with differing soil texture and fertility. Our indicator species analyses demonstrate distinct live and total communities within early growing season soil. Live and total bacterial and fungal communities were significantly influenced by site and crop with all communities showing a slightly higher site effect. Although the live community represented a snapshot of the total community, site and crop-specific species were identified predominately within the live community. Bacterial indicator species were highly diverse with Myxobacteria generally associating with silty switchgrass communities. Mortierellomycota species, which scavenge for phosphorous and associate with mycorrhizal fungi, were indicators of silty soil. Glomeromycota species, which form beneficial associations with rhizomes, were indicative of switchgrass. Our comparison of live and total bacterial and fungal communities residing under switchgrass and maize revealed distinct live communities driven by similar environmental variables. Understanding the effect of agricultural management on live and total communities will further our understanding of how agricultural management and environmental change alter the soil microbiome.

## Data Availability Statement

The datasets presented in this study can be found in online repositories. The names of the repository/repositories and accession number(s) can be found at: https://www.ncbi.nlm.nih.gov/, PRJNA664804.

## Author Contributions

KH and SB designed the experiment. SB collected and analyzed the samples. CK and SL performed data analyses and contributed to the statistical analysis. All authors contributed to the article and approved the submitted version.

## Funding

This research was supported by an Early Career Research Program award to KH, funded by the U.S. Department of Energy, Office of Science, Office of Biological and Environmental Research Genomic Science program under FWP 68292. A portion of this work was performed in the William R. Wiley Environmental Molecular Sciences Laboratory (EMSL), a national scientific user facility sponsored by OBER and located at PNNL. PNNL is a multi-program national laboratory operated by Battelle for the DOE under Contract DE-AC05-76RLO 1830. Support for this research was provided by the Great Lakes Bioenergy Research Center, U.S. Department of Energy, Office of Science, Office of Biological and Environmental Research (Award DE-SC0018409), by the National Science Foundation Long-term Ecological Research Program (DEB 1832042) at the Kellogg Biological Station, and by Michigan State University AgBioResearch.

## Conflict of Interest

The authors declare that the research was conducted in the absence of any commercial or financial relationships that could be construed as a potential conflict of interest.

## Publisher’s Note

All claims expressed in this article are solely those of the authors and do not necessarily represent those of their affiliated organizations, or those of the publisher, the editors and the reviewers. Any product that may be evaluated in this article, or claim that may be made by its manufacturer, is not guaranteed or endorsed by the publisher.
