# Supplementary material for: Site and Bioenergy Cropping System Similarly Affect Distinct Live and Total Soil Microbial Communities
Source: Front Microbiol. 2021 Oct 14;12:725756. doi: 10.3389/fmicb.2021.725756 (PMC8551758; doi:10.3389/fmicb.2021.725756)
Supplement: Supplementary file 5 [file Table_1.docx]

**Supplementary Table 1** Average and standard error (n=20) alpha diversity (Inverse Simpson index) and evenness (Shannon index) of the RNA and DNA fungal and bacterial communities after different normalization methods (cumulative sum scaling normalization or rarefication). Letters denote significance at *p* < 0.05 between normalization methods. Diversity and evenness results were similar between rarified and normalized data. However, the distribution of rare fungal taxa was more evenly distributed when all low abundant species were included; when rarefied, the remaining and more dominant members were less evenly distributed. This is likely driven in part because the bacterial community has greater richness and contains fewer dominant members with lower relative abundances compared to the fungal community. As a result, bacterial evenness is less impacted than the fungal evenness.

| **Normalization** | **Organism** | **Nucleic Acid** | **Measure** | **Average ± SE** |
| --- | --- | --- | --- | --- |
| CSS | bacteria | DNA | invsimpson | 135.3 ± 10.0 ^a^ |
| RAREFY | bacteria | DNA | invsimpson | 134.3 ± 9.8 ^a^ |
| CSS | bacteria | RNA | invsimpson | 471.9 ± 22.2 ^a^ |
| RAREFY | bacteria | RNA | invsimpson | 468.7 ± 22.3 ^a^ |
| CSS | bacteria | DNA | shannon | 0.8 ± 0.0 ^a^ |
| RAREFY | bacteria | DNA | shannon | 0.8 ± 0.0 ^a^ |
| CSS | bacteria | RNA | shannon | 0.9 ± 0.0 ^a^ |
| RAREFY | bacteria | RNA | shannon | 0.9 ± 0.0 ^a^ |
| CSS | fungi | DNA | invsimpson | 25.9 ± 2.7 ^a^ |
| RAREFY | fungi | DNA | invsimpson | 25.9 ± 2.6 ^a^ |
| CSS | fungi | RNA | invsimpson | 21.7 ± 2.2 ^a^ |
| RAREFY | fungi | RNA | invsimpson | 21.6 ± 2.2 ^a^ |
| CSS | fungi | DNA | shannon | 0.7 ± 0.0 ^a^ |
| RAREFY | fungi | DNA | shannon | 0.1 ± 0.0 ^b^ |
| CSS | fungi | RNA | shannon | 0.7 ± 0.0 ^a^ |
| RAREFY | fungi | RNA | shannon | 0.2 ± 0.0 ^b^ |

**Supplementary Table 2** Abundant, normalized live and total fungal species in each soil/cropping type combination.

| **Dominant Live ITS OTUs** | | | | **Dominant Total ITS OTUs** | | | |
| --- | --- | --- | --- | --- | --- | --- | --- |
| **Sandy Maize** | | | | **Sandy Maize** | | | |
| **OTU** | **Taxonomy** | **Similarity** | **Rel. Abund. (Mean ± Stdev)** | **OTU** | **Taxonomy** | **Similarity** | **Rel. Abund. (Mean ± Stdev)** |
| 14 | *Mortierella rishikesha* | 98% | 9.2 ± 7.1% | 15 | *Cystofilobasidium macerans* | 100% | 8.5 ± 11.6% |
| 48 | *Psilocybe flocculosa* | 98% | 6.6 ± 11.2% | 48 | *Psilocybe flocculosa* | 98% | 8.0 ± 10.5% |
| 67 | *Candida restingae* | 100% | 5.1 ± 11.3% | 14 | *Mortierella rishikesha* | 98% | 4.2 ± 3.6% |
| 15 | *Cystofilobasidium macerans* | 100% | 4.9 ± 7.3% | 84 | *Exophiala* sp. | 100% | 3.4 ± 2.2% |
| 66 | *Holtermanniella festucosa* | 100% | 3.7 ± 5.3% | 85 | *Tetracladium* sp. | 94% | 2.7 ± 1.6% |
| **Sandy Switchgrass** | | | | **Sandy Switchgrass** | | | |
| 82 | *Paraglomus laccatum* | 96% | 5.0 ± 5.7% | 31 | *Marasmius rotalis* | 99% | 7.7 ± 13.2% |
| 80 | *Cantharellus hygrophoroides* | 100% | 4.6 ± 3.4% | 30 | *Olpidium* sp. | 100% | 5.5 ± 7.3% |
| 110 | *Septoglomus* sp. | 99% | 3.8 ± 3.1% | 49 | *Pseudopryanochaeta terrestris* | 82% | 4.0 ± 3.0% |
| 193 | *Paraglomus laccatum* | 99% | 3.0 ± 1.4% | 17 | *Phallus rugulosus* | 100% | 3.8 ± 6.1% |
| 86 | *Glomus* sp. | 93% | 2.6 ± 4.1% | 65 | *Ceratobasidium cornigerum* | 90% | 2.9 ± 2.7% |
| **Silty Maize** | | | | **Silty Maize** | | | |
| 90 | *Candida saraburiensis* | 100% | 8.1 ± 18.1% | 33 | *Crepidotus lateralipes* | 87% | 6.4 ± 14.2% |
| 1 | *Mortierella schmuckeri* | 96% | 6.6 ± 5.7% | 21 | *Solicoccozyma terrea* | 100% | 4.1 ± 3.0% |
| 10 | *Mortierella* sp. | 100% | 4.7 ± 6.1% | 50 | *Macgarvieomyces luzulae* | 82% | 3.8 ± 4.1% |
| 6561 | *Uncultured fungus* | 88% | 4.4 ± 9.8% | 1 | *Mortierella schmuckeri* | 96% | 3.7 ± 2.8% |
| 50 | *Uncultured Sordariales* | 97% | 3.9 ± 4.5% | 60 | *Ophiosphaerella* sp. | 99% | 3.6 ± 5.2% |
| **Silty Switchgrass** | | | | **Silty Switchgrass** | | | |
| 23 | *Pleosporales* sp. | 100% | 6.5 ± 2.9% | 34 | *Olpidium brassicae* | 99% | 7.7 ± 17.3% |
| 1 | *Mortierella schmuckeri* | 96% | 4.5 ± 5.0% | 10 | *Mortierella* sp. | 100% | 6.5 ± 12.9% |
| 32 | *Rhizophagus proliferus* | 98% | 3.9 ± 1.8% | 23 | *Pleosporales* sp. | 100% | 6.3 ± 1.2% |
| 4 | *Mortierella hyalina* | 100% | 3.7 ± 4.4% | 11 | *Helotiales* sp. | 96% | 5.5 ± 0.8% |
| 69 | *Funneliformis mosseae* | 77% | 3.4 ± 6.9% | 1 | *Mortierella schmuckeri* | 96% | 3.5 ± 2.1% |

**Supplementary Table 3** Abundant, normalized live and total fungal species in each soil and cropping type.

| **Dominant Live ITS OTUs** | | | | **Dominant Total ITS OTUs** | | | |
| --- | --- | --- | --- | --- | --- | --- | --- |
| **Sandy Soil** | | | | **Sandy Soil** | | | |
| **OTU** | **Taxonomy** | **Similarity** | **Rel. Abund. (Mean ± Stdev)** | **OTU** | **Taxonomy** | **Similarity** | **Rel. Abund. (Mean ± Stdev)** |
| 14 | *Mortierella rishikesha* | 98% | 4.8 ± 6.6% | 48 | *Psilocybe flocculosa* | 98% | 5.3 ± 8.4% |
| 48 | *Psilocybe flocculosa* | 98% | 4.3 ± 8.3% | 15 | *Cystofilobasidium macerans* | 100% | 4.3 ± 8.9% |
| 67 | *Candida restingae* | 100% | 3.4 ± 7.8% | 31 | *Marasmius rotalis* | 99% | 3.9 ± 9.6% |
| 80 | *Cantharellus hygrophoroides* | 100% | 3.1 ± 2.9% | 30 | *Olpidium* sp. | 100% | 3.0 ± 5.6% |
| 86 | *Glomus* sp. | 93% | 2.5 ± 3.6% | 14 | *Mortierella rishikesha* | 98% | 2.5 ± 3.0% |
| **Silty Soil** | | | | **Silty Soil** | | | |
| 1 | *Mortierella schmuckeri* | 96% | 5.5 ± 5.2% | 10 | *Mortierella* sp. | 100% | 4.5 ± 9.0% |
| 90 | *Candida saraburiensis* | 100% | 4.1 ± 12.8% | 23 | *Pleosporales* sp. | 100% | 4.0 ± 2.9% |
| 10 | *Mortierella* sp. | 100% | 3.7 ± 5.0% | 34 | *Olpidium brassicae* | 99% | 3.9 ± 12.2% |
| 23 | *Pleosporales* sp. | 100% | 3.7 ± 3.8% | 21 | *Solicoccozyma terrea* | 100% | 3.8 ± 2.7% |
| 4 | *Mortierella hyalina* | 100% | 3.4 ± 3.6% | 1 | *Mortierella schmuckeri* | 96% | 3.6 ± 2.3% |
| **Maize** | | | | **Maize** | | | |
| 14 | *Mortierella rishikesha* | 98% | 6.1 ± 6.2% | 15 | *Cystofilobasidium macerans* | 100% | 4.2 ± 8.9% |
| 90 | *Candida saraburiensis* | 100% | 4.1 ± 12.8% | 48 | *Psilocybe flocculosa* | 98% | 4.0 ± 8.2% |
| 1 | *Mortierella schmuckeri* | 96% | 3.4 ± 5.1% | 33 | *Crepidotus lateralipes* | 87% | 3.2 ± 10.1% |
| 48 | *Psilocybe flocculosa* | 98% | 3.3 ± 8.3% | 14 | *Mortierella rishikesha* | 98% | 2.8 ± 2.8% |
| 67 | *Candida restingae* | 100% | 2.5 ± 8.0% | 21 | *Solicoccozyma terrea* | 100% | 2.6 ± 2.6% |
| **Switchgrass** | | | | **Switchgrass** | | | |
| 23 | *Pleosporales* sp. | 100% | 3.4 ± 3.8% | 31 | *Marasmius rotalis* | 99% | 4.0 ± 9.6% |
| 110 | *Septoglomus* sp. | 99% | 2.9 ± 2.7% | 34 | *Olpidium brassicae* | 99% | 3.9 ± 12.2% |
| 82 | *Paraglomus laccatum* | 96% | 2.5 ± 4.6% | 23 | *Pleosporales* sp. | 100% | 3.7 ± 2.8% |
| 80 | *Cantharellus hygrophoroides* | 100% | 2.3 ± 3.3% | 10 | *Mortierella* sp. | 100% | 3.3 ± 9.3% |
| 1 | *Mortierella schmuckeri* | 96% | 2.2 ± 4.1% | 11 | *Helotiales* sp. | 96% | 3.2 ± 2.5% |

**Supplementary Table 4** Abundant, normalized live and total fungal indicator species in each soil/cropping type combination.

| **Indicator Species Live ITS OTUs** | | | | | | **Indicator Species Total ITS OTUs** | | | | | |
| --- | --- | --- | --- | --- | --- | --- | --- | --- | --- | --- | --- |
| **Sandy Maize** | | | | | | **Sandy Maize** | | | | | |
| **OTU** | **Taxonomy** | **Similarity** | **Rel. Abund. (Mean ± Stdev)** | **Stat** | **p-value** | **OTU** | **Taxonomy** | **Similarity** | **Rel. Abund. (Mean ± Stdev)** | **Stat** | **p-value** |
| 15 | *Cystofilobasidium macerans* | 96% | 2.6 ± 3.7% | 0.96 | 0.033 | 66 | *Holtermanniella festucosa* | 100% | 2.3 ± 3.1% | 1.00 | 0.002 |
| 250 | *Glomus macrocarpum* | 98% | 1.8 ± 2.6% | 0.84 | 0.046 | 287 | *Septoria protearum* | 98% | 0.8 ± 0.3% | 1.00 | 0.001 |
| 287 | *Septoria protearum* | 98% | 1.0 ± 2.3% | 0.77 | 0.038 | 139 | *Cystofilobasidium macerans* | 99% | 0.5 ± 1.0% | 0.98 | 0.011 |
| 145 | *Calycina alstrupii* | 77% | 0.6 ± 0.5% | 0.83 | 0.033 | 317 | *Septoriella phragmitis* | 100% | 0.4 ± 0.5% | 0.94 | 0.025 |
| 817 | *Leohumicola minimum* | 91% | 0.2 ± 0.2% | 0.77 | 0.042 | 476 | *Conlarium aquaticum* | 95% | 0.4 ± 0.5% | 0.96 | 0.002 |
| **Sandy Switchgrass** | | | | | | **Sandy Switchgrass** | | | | | |
| 82 | *Suhomyces kunorum* | 100% | 5.5 ± 7.4% | 1.00 | 0.001 | 17 | *Phallus mengsongensis* | 97% | 4.5 ± 7.6% | 0.99 | 0.015 |
| 193 | *Suhomyces kunorum* | 100% | 3.2 ± 2.0% | 1.00 | 0.001 | 135 | *Parastagonospora forlicesenica* | 98% | 1.3 ± 1.1% | 0.99 | 0.011 |
| 92 | *Genoderma nasalanense* | 82% | 1.4 ± 2.7% | 0.99 | 0.001 | 92 | *Genoderma nasalanense* | 82% | 0.9 ± 1.2% | 0.89 | 0.005 |
| 222 | *Candida linzhiensis* | 100% | 1.3 ± 2.3% | 0.77 | 0.034 | 215 | *Ceratobasidium papillatum* | 83% | 0.7 ± 0.7% | 1.00 | 0.003 |
| 178 | *Funneliformis mosseae* | 82% | 1.1 ± 0.8% | 0.93 | 0.002 | 333 | *Colletotrichum kakivorum* | 83% | 0.6 ± 0.8% | 0.99 | 0.003 |
| **Silty Maize** | | | | | | **Silty Maize** | | | | | |
| 1927 | *Farysia itapuensis* | 100% | 0.0 ± 0.0% | 0.76 | 0.041 | 1854 | *Kodamaea nitidulidarum* | 91% | 0.5 ± 0.7% | 0.96 | 0.004 |
| NA | NA | NA | NA ± NA% | NA | NA | 595 | *Tubaria recta* | 90% | 0.4 ± 0.2% | 1.00 | 0.004 |
| NA | NA | NA | NA ± NA% | NA | NA | 936 | *Rhizophagus proliferus* | 79% | 0.3 ± 0.2% | 0.91 | 0.004 |
| NA | NA | NA | NA ± NA% | NA | NA | 493 | *Glomus macrocarpum* | 96% | 0.2 ± 0.2% | 0.88 | 0.023 |
| NA | NA | NA | NA ± NA% | NA | NA | 606 | *Solicoccozyma aeria* | 100% | 0.2 ± 0.3% | 0.77 | 0.025 |
| **Silty Switchgrass** | | | | | | **Silty Switchgrass** | | | | | |
| 63 | *Pholiotina mediterranea* | 89% | 2.9 ± 4.4% | 0.85 | 0.013 | 211 | *Funneliformis mosseae* | 98% | 0.6 ± 0.7% | 0.83 | 0.046 |
| 69 | *Funneliformis mosseae* | 77% | 2.3 ± 4.7% | 0.77 | 0.045 | 117 | *Chalara pseudoaffinis* | 94% | 0.5 ± 0.7% | 0.88 | 0.027 |
| 159 | *Funneliformis mosseae* | 97% | 1.2 ± 1.4% | 0.99 | 0.003 | 477 | *Coprinopsis scobicola* | 91% | 0.4 ± 0.3% | 0.96 | 0.002 |
| 2178 | *Tremella* sp. | 100% | 1.1 ± 1.3% | 0.77 | 0.037 | 475 | *Monascella botryosa* | 87% | 0.3 ± 0.5% | 0.77 | 0.027 |
| 16 | *Diversispora spurca* | 100% | 0.7 ± 1.4% | 0.77 | 0.033 | 39 | *Alpinaria rhododendri* | 96% | 0.3 ± 0.3% | 0.97 | 0.011 |

**Supplementary Table 5** Abundant, normalized live and total fungal indicator species in each soil and cropping type.

| **Indicator Species Live ITS OTUs** | | | | | | **Indicator Species Total ITS OTUs** | | | | | |
| --- | --- | --- | --- | --- | --- | --- | --- | --- | --- | --- | --- |
| **Sandy Soil** | | | | | | **Sandy Soil** | | | | | |
| **OTU** | **Taxonomy** | **Similarity** | **Rel. Abund. (Mean ± Stdev)** | **Stat** | **p-value** | **OTU** | **Taxonomy** | **Similarity** | **Rel. Abund. (Mean ± Stdev)** | **Stat** | **p-value** |
| 80 | *Cantharellus hygrophoroides* | 100% | 3.1 ± 2.9% | 0.89 | 0.011 | 15 | *Cystofilobasidium macerans* | 100% | 4.3 ± 8.9% | 1.00 | 0.001 |
| 5206 | *Glomeraceae* sp. | 88% | 0.0 ± 0.0% | 0.77 | 0.038 | 35 | *Cladosporium angustiherbarum* | 100% | 2.0 ± 2.8% | 0.99 | 0.001 |
| NA | NA | NA | NA | NA | NA | 49 | *Pseudopyrenocaeta terrestris* | 82% | 2.2 ± 2.8% | 0.99 | 0.002 |
| NA | NA | NA | NA | NA | NA | 73 | *Fusarium petersiae* | 100% | 1.3 ± 2.0% | 0.99 | 0.001 |
| NA | NA | NA | NA | NA | NA | 85 | *Tetracladium* sp. | 94% | 1.4 ± 1.8% | 1.00 | 0.001 |
| **Silty Soil** | | | | | | **Silty Soil** | | | | | |
| 1 | *Mortierella schmuckeri* | 96% | 5.5 ± 5.2% | 1.00 | 0.001 | 10 | *Mortierella* sp. | 100% | 4.5 ± 9.0% | 0.99 | 0.001 |
| 10 | *Mortierella* sp. | 100% | 3.7 ± 5.0% | 1.00 | 0.001 | 23 | *Pleosporales* sp. | 100% | 4.0 ± 2.9% | 0.94 | 0.013 |
| 23 | *Pleosporales* sp. | 100% | 3.7 ± 3.8% | 0.99 | 0.014 | 21 | *Solicoccozyma terrea* | 100% | 3.8 ± 2.7% | 0.91 | 0.005 |
| 4 | *Mortierella hyalina* | 100% | 3.4 ± 3.6% | 1.00 | 0.001 | 1 | *Mortierella schmuckeri* | 96% | 3.6 ± 2.3% | 0.98 | 0.001 |
| 34 | *Olpidium brassicae* | 99% | 0.5 ± 1.5% | 0.99 | 0.033 | 11 | *Helotiales* sp. | 96% | 3.4 ± 2.4% | 0.92 | 0.025 |
| **Maize** | | | | | | **Maize** | | | | | |
| NA | NA | NA | NA | NA | NA | 84 | *Exophiala* sp. | 100% | 2.6 ± 1.9% | 0.99 | 0.002 |
| NA | NA | NA | NA | NA | NA | 134 | *Schizothecium carpinicola* | 84% | 1.5 ± 2.0% | 0.84 | 0.020 |
| NA | NA | NA | NA | NA | NA | 3 | *Fusarium pseudoanthophilium* | 100% | 1.2 ± 0.4% | 0.79 | 0.028 |
| NA | NA | NA | NA | NA | NA | 2127 | *Penicillifer* sp. | 99% | 1.2 ± 1.1% | 0.96 | 0.003 |
| NA | NA | NA | NA | NA | NA | 119 | *Leohumicola minima* | 90% | 0.5 ± 1.3% | 0.99 | 0.003 |
| **Switchgrass** | | | | | | **Switchgrass** | | | | | |
| 110 | *Septoglomus* sp. | 100% | 2.9 ± 2.7% | 0.89 | 0.044 | 30 | *Hannaella pagnoccae* | 100% | 3.1 ± 5.5% | 0.96 | 0.025 |
| 225 | *Glomus* sp. | 97% | 1.6 ± 1.6% | 0.93 | 0.016 | 124 | *Sordariomycetes* sp. | 94% | 1.4 ± 1.4% | 0.88 | 0.012 |
| 6169 | *Archaeospora ecuadoriana* | 100% | 1.4 ± 1.6% | 0.99 | 0.002 | 161 | *Leohumicola minima* | 93% | 0.9 ± 0.7% | 0.93 | 0.011 |
| 262 | *Septoglomus* sp. | 98% | 1.4 ± 1.3% | 0.86 | 0.011 | 99 | *Hymenoscyphus* sp. | 100% | 0.8 ± 1.0% | 0.98 | 0.006 |
| 205 | *Glomus macrocarpum* | 93% | 0.8 ± 1.0% | 0.89 | 0.021 | 32 | *Rhizophagus proliferus* | 80% | 0.8 ± 0.5% | 0.89 | 0.025 |

**Supplementary Table 6** Abundant, normalized live and total bacterial species in each soil/cropping type combination.

| **Dominant Live 16S OTUs** | | | | **Dominant Total 16S OTUs** | | | |
| --- | --- | --- | --- | --- | --- | --- | --- |
| **Sandy Maize** | | | | **Sandy Maize** | | | |
| **OTU** | **Taxonomy** | **Similarity** | **Rel. Abund. (Mean ± Stdev)** | **OTU** | **Taxonomy** | **Similarity** | **Rel. Abund. (Mean ± Stdev)** |
| 71 | *Nitrosospira* sp. | 90% | 1.1 ± 0.2% | 22 | *Chthiobacter flavus* | 92% | 5.9 ± 1.7% |
| 49 | *Luteitalea pratensis* | 96% | 0.9 ± 0.2% | 55 | *Brevitalea* sp. | 94% | 3.5 ± 0.9% |
| 54 | *Silvibacterium bohemicum* | 93% | 0.9 ± 0.4% | 48 | *Nitrososphaera viennensis* | 95% | 1.1 ± 0.6% |
| 52 | *Haliangium ochraceum* | 91% | 0.9 ± 0.3% | 84 | *Luteibacter anthropi* | 86% | 1.1 ± 0.2% |
| 96 | *Acidobacterium ailaaui* | 91% | 0.7 ± 0.3% | 65 | *Rhodoplanes piscinae* | 98% | 1.1 ± 0.2% |
| **Sandy Switchgrass** | | | | **Sandy Switchgrass** | | | |
| 52 | *Haliangium ochraceum* | 91% | 1.7 ± 0.6% | 22 | *Chthiobacter flavus* | 92% | 6.2 ± 1.1% |
| 2331 | *Piscinibacter aquaticus* | 99% | 1.0 ± 0.3% | 55 | *Brevitalea* sp. | 94% | 3.2 ± 0.5% |
| 49 | *Luteitalea pratensis* | 96% | 1.0 ± 0.3% | 58 | *Brevitalea* sp. | 93% | 2.0 ± 0.2% |
| 250 | *Rhizobacter* sp. | 100% | 0.8 ± 0.1% | 89 | *Chthoniobacter flavus* | 91% | 1.3 ± 0.1% |
| 71 | *Nitrosospira* sp. | 90% | 0.8 ± 0.4% | 49 | *Luteitalea pratensis* | 96% | 1.0 ± 0.2% |
| **Silty Maize** | | | | **Silty Maize** | | | |
| 52 | *Haliangium ochraceum* | 91% | 1.6 ± 0.6% | 22 | *Chthiobacter flavus* | 92% | 9.7 ± 5.4% |
| 49 | *Luteitalea pratensis* | 96% | 0.7 ± 0.1% | 55 | *Brevitalea* sp. | 94% | 1.5 ± 1.1% |
| 73 | *Limisphaera ngatamarikiensis* | 80% | 0.7 ± 0.1% | 89 | *Chthoniobacter flavus* | 91% | 1.3 ± 0.4% |
| 93 | *Cystobacter gracilis* | 98% | 0.7 ± 0.3% | 84 | *Luteibacter anthropi* | 86% | 1.3 ± 0.5% |
| 220 | *Kofleria flava* | 91% | 0.6 ± 0.3% | 65 | *Rhodoplanes piscinae* | 98% | 1.2 ± 0.2% |
| **Silty Switchgrass** | | | | **Silty Switchgrass** | | | |
| 52 | *Haliangium ochraceum* | 91% | 1.9 ± 0.3% | 22 | *Chthiobacter flavus* | 92% | 6.6 ± 1.8% |
| 25 | *Aeromonas* sp. | 100% | 1.2 ± 2.7% | 41 | *Niabella terrea* | 97% | 2.0 ± 0.5% |
| 2 | *Pseudomonas* sp. | 100% | 1.1 ± 2.1% | 48 | *Nitrososphaera viennensis* | 95% | 1.4 ± 0.5% |
| 73 | *Limisphaera ngatamarikiensis* | 80% | 1.0 ± 0.2% | 89 | *Chthoniobacter flavus* | 91% | 1.3 ± 0.2% |
| 340 | *Thermodesulfovibrio hydrogeniphilus* | 80% | 0.9 ± 0.2% | 49 | *Luteitalea pratensis* | 96% | 1.2 ± 0.1% |

**Supplementary Table 7** Abundant, normalized live and total bacterial species in each soil and cropping type.

| **Dominant Live 16S OTUs** | | | | **Dominant Total 16S OTUs** | | | |
| --- | --- | --- | --- | --- | --- | --- | --- |
| **Sandy Soil** | | | | **Sandy Soil** | | | |
| **OTU** | **Taxonomy** | **Similarity** | **Rel. Abund. (Mean ± Stdev)** | **OTU** | **Taxonomy** | **Similarity** | **Rel. Abund. (Mean ± Stdev)** |
| 52 | *Haliangium ochraceum* | 91% | 1.3 ± 0.6% | 22 | *Chthiobacter flavus* | 92% | 6.1 ± 1.3% |
| 71 | *Nitrosospira* sp. | 90% | 1.0 ± 0.3% | 55 | *Brevitalea* sp. | 94% | 3.3 ± 0.7% |
| 49 | *Luteitalea pratensis* | 96% | 0.9 ± 0.2% | 58 | *Brevitalea* sp. | 93% | 1.5 ± 0.6% |
| 9540 | *Paludibaculum fermentans* | 90% | 0.7 ± 0.3% | 89 | *Chthoniobacter flavus* | 91% | 1.2 ± 0.3% |
| 2331 | *Piscinibacter aquaticus* | 99% | 0.7 ± 0.4% | 84 | *Luteibacter anthropi* | 86% | 1.0 ± 0.2% |
| **Silty Soil** | | | | **Silty Soil** | | | |
| 52 | *Haliangium ochraceum* | 91% | 1.7 ± 0.5% | 22 | *Chthiobacter flavus* | 92% | 8.2 ± 4.2% |
| 73 | *Limisphaera ngatamarikiensis* | 80% | 0.8 ± 0.2% | 41 | *Niabella terrea* | 97% | 1.5 ± 0.8% |
| 49 | *Luteitalea pratensis* | 96% | 0.8 ± 0.1% | 55 | *Brevitalea* sp. | 94% | 1.4 ± 0.8% |
| 340 | *Thermodesulfovibrio hydrogeniphilus* | 80% | 0.7 ± 0.2% | 89 | *Chthoniobacter flavus* | 91% | 1.3 ± 0.3% |
| 25 | *Aeromonas* sp. | 100% | 0.6 ± 1.9% | 84 | *Luteibacter anthropi* | 86% | 1.2 ± 0.4% |
| **Maize** | | | | **Maize** | | | |
| 52 | *Haliangium ochraceum* | 91% | 1.2 ± 0.5% | 22 | *Chthiobacter flavus* | 92% | 7.8 ± 4.3% |
| 49 | *Luteitalea pratensis* | 96% | 0.8 ± 0.2% | 55 | *Brevitalea* sp. | 94% | 2.5 ± 1.4% |
| 54 | *Silvibacterium bohemicum* | 93% | 0.7 ± 0.6% | 84 | *Luteibacter anthropi* | 86% | 1.2 ± 0.4% |
| 9540 | *Paludibaculum fermentans* | 90% | 0.6 ± 0.2% | 89 | *Chthoniobacter flavus* | 91% | 1.2 ± 0.4% |
| 71 | *Nitrosospira* sp. | 90% | 0.6 ± 0.5% | 65 | *Rhodoplanes piscinae* | 98% | 1.1 ± 0.2% |
| **Switchgrass** | | | | **Switchgrass** | | | |
| 52 | *Haliangium ochraceum* | 91% | 1.8 ± 0.5% | 22 | *Chthiobacter flavus* | 92% | 6.4 ± 1.4% |
| 49 | *Luteitalea pratensis* | 96% | 0.9 ± 0.2% | 55 | *Brevitalea* sp. | 94% | 2.2 ± 1.1% |
| 2 | *Pseudomonas* sp. | 100% | 0.8 ± 1.5% | 89 | *Chthoniobacter flavus* | 91% | 1.3 ± 0.2% |
| 2331 | *Piscinibacter aquaticus* | 99% | 0.7 ± 0.4% | 58 | *Brevitalea* sp. | 93% | 1.3 ± 0.8% |
| 250 | *Rhizobacter* sp. | 100% | 0.7 ± 0.2% | 41 | *Niabella terrea* | 97% | 1.3 ± 0.9% |

| **Indicator Species Live 16S OTUs** | | | | | | **Indicator Species Total 16S OTUs** | | | | | |
| --- | --- | --- | --- | --- | --- | --- | --- | --- | --- | --- | --- |
| **Sandy Maize** | | | | | | **Sandy Maize** | | | | | |
| **OTU** | **Taxonomy** | **Similarity** | **Rel. Abund. (Mean ± Stdev)** | **Stat** | **p-value** | **OTU** | **Taxonomy** | **Similarity** | **Rel. Abund. (Mean ± Stdev)** | **Stat** | **p-value** |
| 222 | *Acidibacter ferrireducens* | 96% | 0.3 ± 0.2% | 0.98 | 0.001 | 51 | *Nitrososphaera viennensis* | 91% | 0.7 ± 0.6% | 0.98 | 0.001 |
| 95 | *Paludibaculum fermentans* | 92% | 0.2 ± 0.3% | 1.00 | 0.001 | 637 | *Occallatibacter riparius* | 93% | 0.2 ± 0.2% | 0.94 | 0.012 |
| 444 | *Actinoallomurus iriomotensis* | 96% | 0.2 ± 0.2% | 0.94 | 0.009 | 255 | *Acidobacterium ailaaui* | 94% | 0.1 ± 0.2% | 0.99 | 0.002 |
| 1106 | *Acidibacter ferrireducens* | 96% | 0.2 ± 0.1% | 0.91 | 0.005 | 46 | *Tepidisphaera mucosa* | 88% | 0.1 ± 0.1% | 0.98 | 0.001 |
| 51 | *Nitrososphaera viennensis* | 91% | 0.1 ± 0.1% | 0.96 | 0.002 | 222 | *Acidibacter ferrireducens* | 96% | 0.1 ± 0.1% | 0.99 | 0.001 |
| **Sandy Switchgrass** | | | | | | **Sandy Switchgrass** | | | | | |
| 764 | *Ktedonobacter racemifer* | 89% | 0.1 ± 0.1% | 0.98 | 0.003 | 441 | *Mangrovitalea sediminis* | 92% | 0.0 ± 0.0% | 0.96 | 0.001 |
| 495 | *Sulfuricaulis limicola* | 94% | 0.1 ± 0.1% | 0.97 | 0.002 | 901 | *Acidobacterium ailaaui* | 94% | 0.0 ± 0.0% | 0.87 | 0.011 |
| 1307 | *Chondromyces robustus* | 88% | 0.1 ± 0.0% | 0.84 | 0.046 | 665 | *Terrimicrobium sacchariphilum* | 86% | 0.0 ± 0.0% | 0.97 | 0.001 |
| 665 | *Terrimicrobium sacchariphilum* | 86% | 0.1 ± 0.1% | 0.98 | 0.001 | 5 | *Acinetobacter calcoaceticus* | 100% | 0.0 ± 0.1% | 0.93 | 0.007 |
| 1368 | *Frankia inefficax* | 97% | 0.0 ± 0.0% | 1.00 | 0.001 | 764 | *Ktedonobacter racemifer* | 89% | 0.0 ± 0.0% | 0.99 | 0.001 |
| **Silty Maize** | | | | | | **Silty Maize** | | | | | |
| 419 | *Ohtaekwangia koreensis* | 96% | 0.1 ± 0.1% | 0.95 | 0.005 | 15210 | *Sunxiuqinia faeciviva* | 83% | 0.0 ± 0.0% | 0.99 | 0.001 |
| 668 | *Thauera propionica* | 96% | 0.0 ± 0.1% | 0.77 | 0.036 | 437 | *Glutamicibacter soli* | 78% | 0.0 ± 0.0% | 0.75 | 0.046 |
| 3816 | *Aggregicoccus edonensis* | 96% | 0.0 ± 0.0% | 0.90 | 0.007 | 4756 | *Chthoniobacter flavus* | 91% | 0.0 ± 0.0% | 0.89 | 0.018 |
| 1154 | *Gimesia maris* | 81% | 0.0 ± 0.0% | 0.86 | 0.006 | 3757 | *Anaeromyxobacter dehalogenans* | 90% | 0.0 ± 0.0% | 0.88 | 0.005 |
| 11080 | *Cystobacter gracilis* | 98% | 0.0 ± 0.0% | 0.84 | 0.008 | 1585 | *Gemmatimonas aurantiaca* | 89% | 0.0 ± 0.0% | 0.89 | 0.019 |
| **Silty Switchgrass** | | | | | | **Silty Switchgrass** | | | | | |
| 385 | *Kofleria flava* | 92% | 0.1 ± 0.1% | 0.94 | 0.001 | 102 | *Streptomyces fukangensis* | 84% | 0.4 ± 0.2% | 0.95 | 0.021 |
| 214 | *Chondromyces robustus* | 92% | 0.1 ± 0.2% | 0.98 | 0.004 | 386 | *Gemmata massiliana* | 84% | 0.2 ± 0.1% | 0.94 | 0.002 |
| 530 | *Chondromyces robustus* | 93% | 0.1 ± 0.0% | 0.96 | 0.001 | 1206 | *Marimicrobium arenosum* | 96% | 0.1 ± 0.0% | 0.87 | 0.047 |
| 607 | *Kofleria flava* | 91% | 0.1 ± 0.0% | 0.92 | 0.006 | 1074 | *Methylosinus trichosporium* | 82% | 0.1 ± 0.0% | 0.84 | 0.035 |
| 587 | *Rubrobacter taiwanensis* | 83% | 0.1 ± 0.1% | 0.90 | 0.009 | 499 | *Salinispirillum marinum* | 82% | 0.0 ± 0.0% | 0.89 | 0.007 |

**Supplementary Table 8** Abundant, normalized live and total bacterial indicator species in each soil/cropping type combination.

**Supplementary Table 9** Abundant, normalized live and total bacterial indicator species in each soil and cropping type.

| **Indicator Species Live 16S OTUs** | | | | | | **Indicator Species Total 16S OTUs** | | | | | |
| --- | --- | --- | --- | --- | --- | --- | --- | --- | --- | --- | --- |
| **Sandy Soil** | | | | | | **Sandy Soil** | | | | | |
| **OTU** | **Taxonomy** | **Similarity** | **Rel. Abund. (Mean ± Stdev)** | **Stat** | **p-value** | **OTU** | **Taxonomy** | **Similarity** | **Rel. Abund. (Mean ± Stdev)** | **Stat** | **p-value** |
| 71 | *Nitrosospira* sp. | 90% | 1.0 ± 0.3% | 0.92 | 0.002 | 55 | *Brevitalea* sp. | 94% | 3.3 ± 0.7% | 0.82 | 0.005 |
| 9540 | *Paludibaculum fermentans* | 90% | 0.7 ± 0.3% | 0.79 | 0.032 | 58 | *Brevitalea* sp. | 93% | 1.5 ± 0.6% | 0.84 | 0.007 |
| 519 | *Paludibaculum fermentans* | 90% | 0.5 ± 0.2% | 0.82 | 0.005 | 80 | *Thermanaerovibrio acidaminovoran* | 85% | 0.6 ± 0.3% | 0.87 | 0.003 |
| 272 | *Leptothrix discophora* | 99% | 0.5 ± 0.2% | 0.81 | 0.006 | 201 | *Sphingomonas* sp. | 100% | 0.6 ± 0.1% | 0.83 | 0.003 |
| 242 | *Paludibaculum fermentans* | 87% | 0.5 ± 0.2% | 0.80 | 0.040 | 71 | *Nitrosospira* sp. | 90% | 0.6 ± 0.3% | 0.92 | 0.001 |
| **Silty Soil** | | | | | | **Silty Soil** | | | | | |
| 73 | *Limisphaera ngatamarikiensis* | 80% | 0.8 ± 0.2% | 0.86 | 0.002 | 41 | *Niabella terrea* | 97% | 1.5 ± 0.8% | 0.89 | 0.005 |
| 340 | *Thermodesulfovibrio hydrogeniphilus* | 80% | 0.7 ± 0.2% | 0.81 | 0.003 | 49 | *Luteitalea pratensis* | 96% | 1.1 ± 0.3% | 0.77 | 0.031 |
| 93 | *Cystobacter gracilis* | 98% | 0.6 ± 0.2% | 0.83 | 0.017 | 67 | *Nitrospira japonica* | 100% | 1.0 ± 0.3% | 0.83 | 0.003 |
| 87 | *Bellilinea* sp. | 95% | 0.6 ± 0.3% | 0.94 | 0.002 | 158 | *Gemmatimonas* sp. | 100% | 0.7 ± 0.4% | 0.88 | 0.003 |
| 41 | *Niabella terrea* | 97% | 0.5 ± 0.3% | 0.87 | 0.006 | 130 | *Chitinophaga* sp. | 100% | 0.6 ± 0.2% | 0.90 | 0.001 |
| **Maize** | | | | | | **Maize** | | | | | |
| 54 | *Silvibacterium bohemicum* | 93% | 0.7 ± 0.6% | 0.95 | 0.004 | 65 | *Rhodoplanes piscinae* | 98% | 1.1 ± 0.2% | 0.78 | 0.001 |
| 91 | *Limisphaera ngatamarikiensis* | 89% | 0.6 ± 0.3% | 0.83 | 0.024 | 78 | *Granulicella sapmiensis* | 91% | 0.8 ± 0.4% | 0.85 | 0.011 |
| 96 | *Acidobacterium ailaaui* | 91% | 0.5 ± 0.4% | 0.89 | 0.012 | 96 | *Acidobacterium ailaaui* | 91% | 0.6 ± 0.4% | 0.86 | 0.03 |
| 109 | *Aetherobacter rufus* | 94% | 0.4 ± 0.2% | 0.80 | 0.012 | 54 | *Silvibacterium bohemicum* | 93% | 0.4 ± 0.3% | 0.92 | 0.019 |
| 61 | *Chujaibacter soli* | 99% | 0.4 ± 0.5% | 1.00 | 0.001 | 106 | *Stenotrophobacter roseus* | 98% | 0.2 ± 0.2% | 0.88 | 0.042 |
| **Switchgrass** | | | | | | **Switchgrass** | | | | | |
| 2 | *Pseudomonas silesiensis* | 100% | 0.8 ± 1.5% | 0.93 | 0.025 | 52 | *Haliangium ochraceum* | 91% | 0.3 ± 0.1% | 0.81 | 0.001 |
| 2331 | *Piscinibacter aquaticus* | 99% | 0.7 ± 0.4% | 0.79 | 0.033 | 417 | *Vicinamibacter silvestris* | 92% | 0.2 ± 0.1% | 0.81 | 0.037 |
| 250 | *Rhizobacter profundi* | 100% | 0.7 ± 0.2% | 0.83 | 0.001 | 787 | *Pedosphaera* sp. | 98% | 0.2 ± 0.0% | 0.80 | 0.003 |
| 204 | *Minicystic rosea* | 95% | 0.5 ± 0.3% | 0.80 | 0.033 | 719 | *Thiohalospira* sp. | 100% | 0.1 ± 0.0% | 0.80 | 0.034 |
| 151 | *Haliangium ochraceum* | 89% | 0.4 ± 0.1% | 0.87 | 0.001 | 184 | *Ancylobacter pratisalsi* | 97% | 0.1 ± 0.0% | 0.85 | 0.011 |

**Supplementary Table 10**Average and standard error (n=5) of microbial biomass carbon (MBC), microbial biomass nitrogen (MBN), MBC:MBN ratio, salt-extractable dissolved organic C (DOC), total dissolved N (TDN), and DOC:TDN ratio from soils sampled. Means with the same letter for a given site/crop combination are not significantly different at *p* < 0.05.

| **Site** | **Crop** | **MBC (mg C g-1 dry soil)** | **MBN (mg N g-1 dry soil)** | **MBC:MBN ratio** | **DOC (mg C g-1 dry soil)** | **TDN (mg N g-1 dry soil)** | **DOC:TDN ratio** |
| --- | --- | --- | --- | --- | --- | --- | --- |
| Sandy | Maize | 118.2 ± 10.2 ^a^ | 12.2 ± 0.8 ^a^ | 9.7 ± 0.9 ^ac^ | 64.2 ± 5.8 ^a^ | 13.9 ± 5.1 ^bc^ | 5.1 ± 1.8 ^ad^ |
| Sandy | Switchgrass | 120.6 ± 17.8 ^a^ | 14.7 ± 1.7 ^a^ | 8.2 ± 0.9 ^ad^ | 56.8 ± 5.6 ^a^ | 8.3 ± 0.8 ^bd^ | 6.8 ± 1.0^ac^ |
| Silty | Maize | 103.0 ± 56.9 ^a^ | 18.0 ± 7.5 ^a^ | 5.5 ± 1.1 ^bc^ | 49.1 ± 14.4 ^b^ | 22.5 ± 7.2 ^ac^ | 2.3 ± 0.5 ^bd^ |
| Silty | Switchgrass | 93.6 ± 61.3 ^a^ | 19.4 ± 10.3 ^a^ | 4.7 ± 1.7 ^bd^ | 46.6 ± 11.1 ^b^ | 15.7 ± 4.2 ^ad^ | 3.0 ± 0.1 ^bc^ |

**
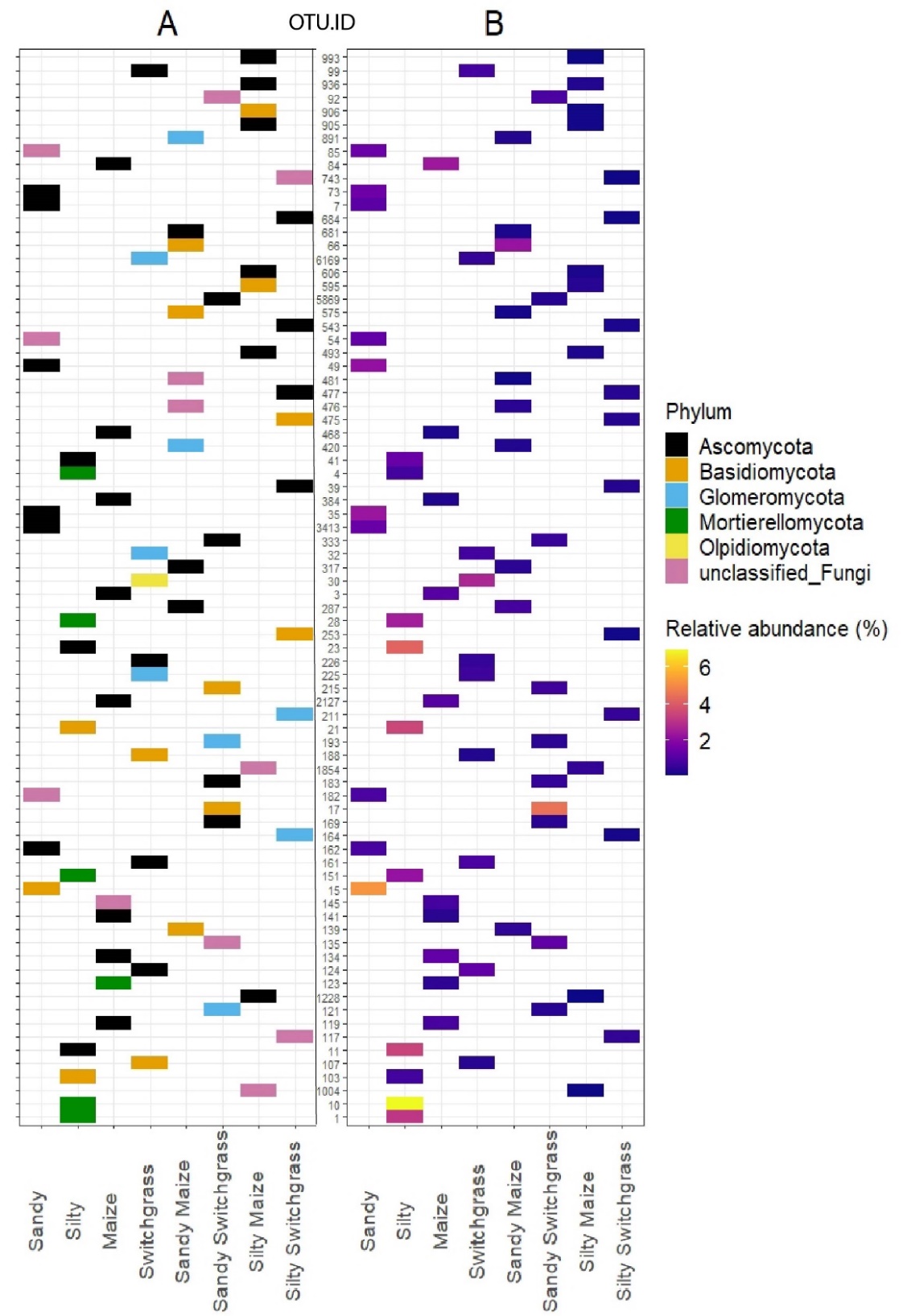
**

**Supplementary Figure 1** Schematic representation of the top ten DNA fungal indicator species within each site, crop, and site/crop combination which can be found within Supplementary File 3. Indicator species are represented as a heatmap matrix colored by phylum (A) and average relative abundance (%) (B). OTU were selected by significance (α<0.05) and indicator value (IV>0.7) for a specific crop or site.

**
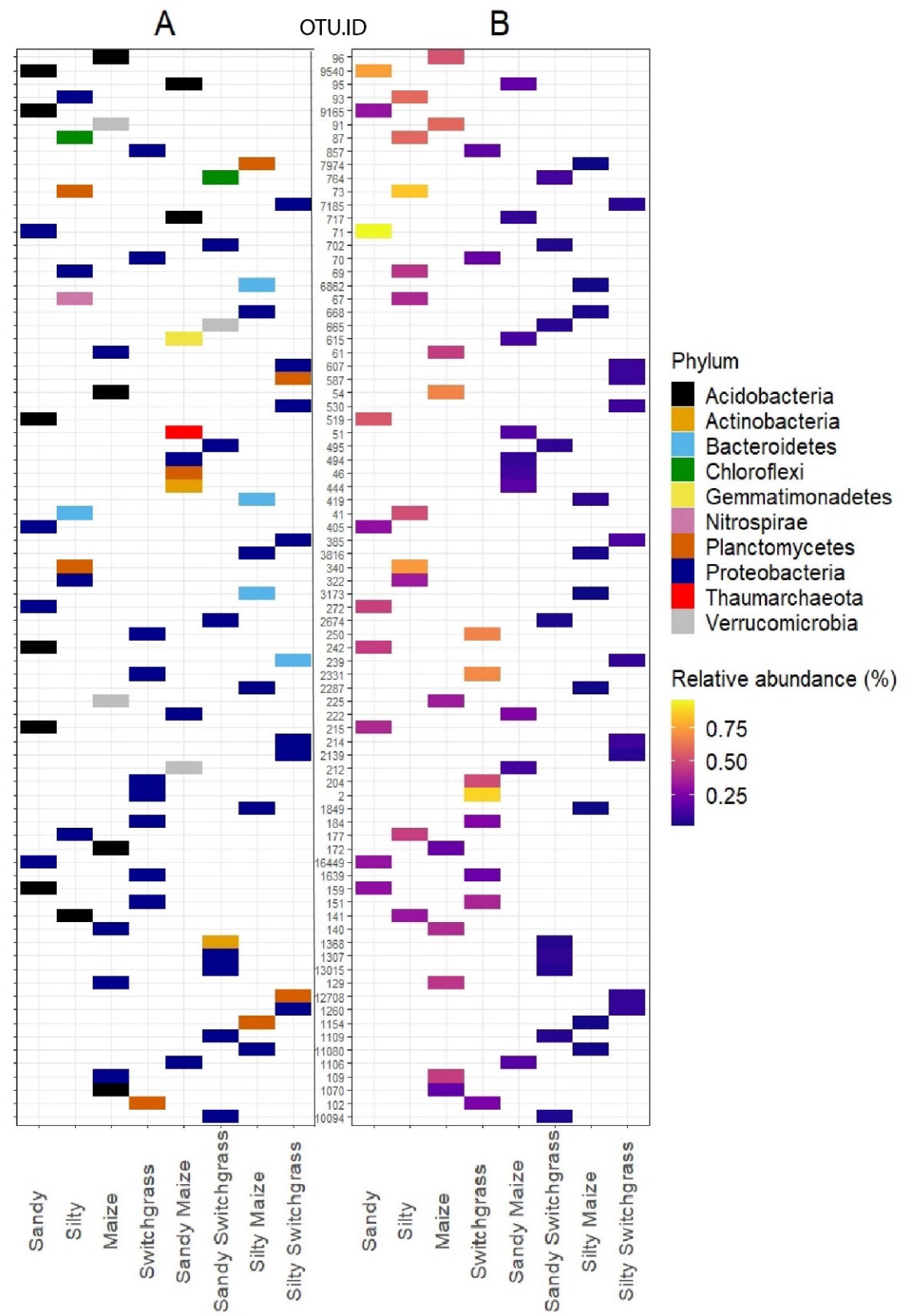
**

**Supplementary Figure 2** Schematic representation of the top ten RNA bacterial indicator species within each site, crop, and site/crop combination which can be found within Supplementary File 4. Indicator species are represented as a heatmap matrix colored by phylum (A) and average relative abundance (%) (B). OTU were selected by significance (α<0.05) and indicator value (IV>0.7) for a specific crop or site.


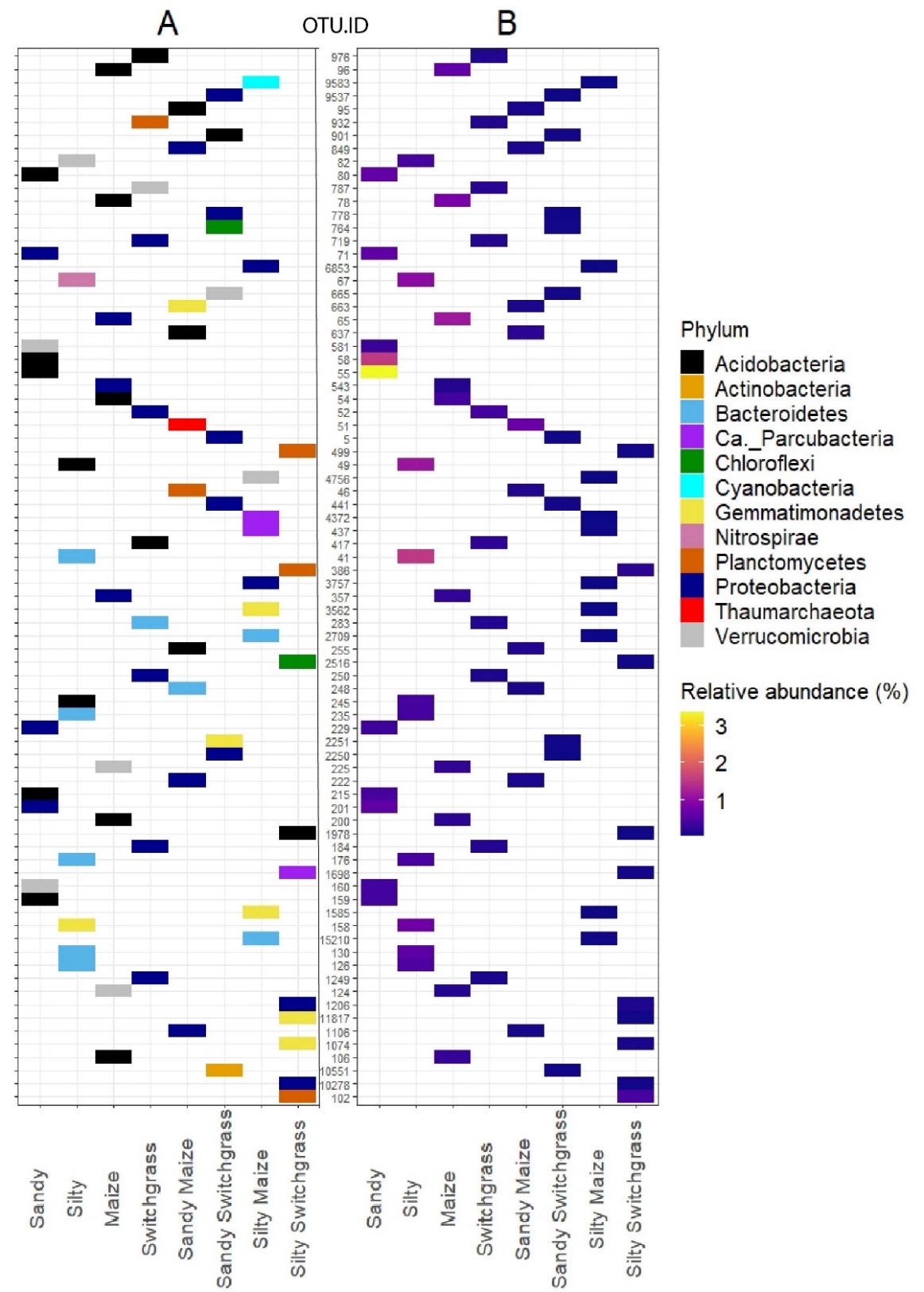


**Supplementary Figure 3** Schematic representation of the top ten DNA bacterial indicator species within each site, crop, and site/crop combination which can be found within Supplementary File 5. Indicator species are represented as a heatmap matrix colored by phylum (A) and average relative abundance (%) (B). OTU were selected by significance (α<0.05) and indicator value (IV>0.7) for a specific crop or site.

**
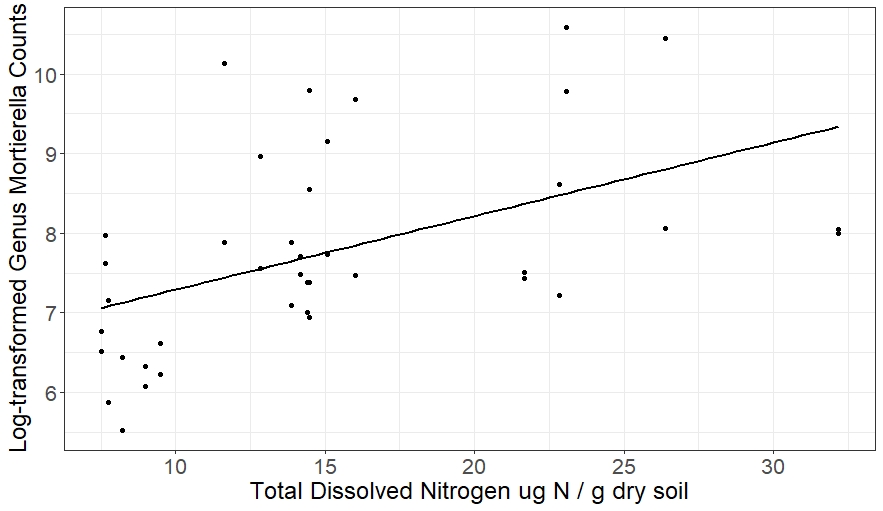
**

R^2^ = 0.22, p=0.001

**Supplementary Figure 4** Linear regression showing the relationship between total dissolved nitrogen and Mortierellomycota genera Mortierella within the fungal community across silty and sandy soils.

**
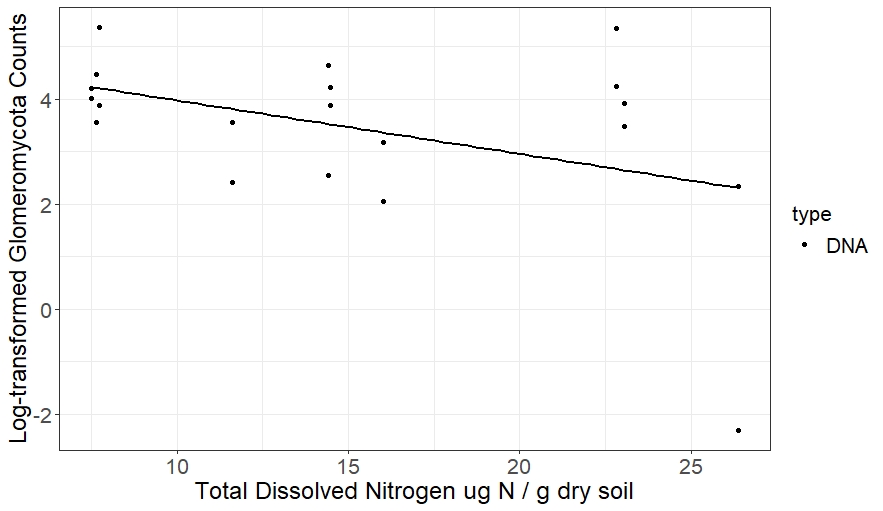
**

R^2^ = 0.13, p=0.065

**Supplementary Figure 5** Linear regression showing the relationship between total dissolved nitrogen and Glomeromycota genera Rhizophagus within the total fungal community across silty and sandy soils.
